# Associations of lung function impairment and biological aging with mortality and cardiovascular disease incidence: findings from UK biobank participants

**DOI:** 10.3389/fpubh.2025.1635195

**Published:** 2025-07-10

**Authors:** Jiali Chen, Cheng Wang, Pengyu Duan, Yonghong Bi, Yao Meng, Weiyu Feng, Zhehao Jin, Hangbing Li, Huihui Wang, Xiaoyan Li, Kun Zuo, Xiangcheng Zhao, Longfei Li, Yuling Xing, Lan Luo, Yang Yu, Miao Yu, Muyan Cui, Bing Zhang

**Affiliations:** ^1^Department of Anesthesiology, The Second Affiliated Hospital of Harbin Medical University, Harbin, China; ^2^Department of Environmental Hygiene, School of Public Health, Harbin Medical University, Harbin, China

**Keywords:** biological aging, cardiovascular disease, frailty, mortality, preserved ratio impaired spirometry (PRISm), phenotypic age acceleration, airflow limitation

## Abstract

**Background:**

Lung function impairment, including preserved ratio impaired spirometry (PRISm) and airflow limitation (AL), is associated with increased risks of mortality and cardiovascular disease; however, the underlying biological mechanisms remain poorly understood. This study aimed to investigate the mediating role of biological aging, as well as the interactions and joint effects of lung function impairment and biological aging on adverse health outcomes.

**Methods:**

A total of 349,456 UK Biobank participants (aged 40–69 years) were categorized into normal, PRISm, and AL groups based on baseline spirometric measurements. Biological aging was assessed using phenotypic age acceleration (PhenoAgeAccel) and frailty phenotype. PhenoAgeAccel was calculated using algorithms based on clinical biomarkers, and frailty was evaluated according to five established criteria. Cox proportional hazards models were used to assess the risks of all-cause mortality and CVD (including coronary artery disease, heart failure, and ischemic stroke). Mediation and interaction analyses further examined the associations between lung function impairment, biological aging, and adverse health outcomes.

**Results:**

Over an average follow-up of 13.8–14.4 years, 28,059 deaths and 34,863 incident CVD cases were recorded. PhenoAgeAccel mediated 12.8–16.2% of the association between lung function impairment and mortality, compared to 7.0–12.0% for frailty. For CVD, the mediation proportions were 8.5–15.9% (PhenoAgeAccel) and 7.4–8.6% (frailty). Significant multiplicative and additive interactions were found between lung function impairment and biological aging, especially for mortality risk. The joint analysis revealed that participants with both impaired lung function and accelerated biological aging had the highest mortality and CVD risks.

**Conclusion:**

Biological aging partially mediates the associations between lung function impairment and adverse outcomes, exhibiting synergistic interactions with impaired lung function. Thus, therapies targeting biological aging may complement conventional lung function promotion in reducing health disparities among aging populations.

## 1 Introduction

Lung function impairment represents a significant global public health challenge, substantially contributing to increased disease burden and mortality rates across populations (1). Airflow limitation (AL), a defining characteristic of chronic obstructive pulmonary disease (COPD), has traditionally been recognized as the primary phenotype of lung function impairment. Recently, the identification of preserved ratio impaired spirometry (PRISm) as a distinct phenotype has generated significant attention in respiratory research (2, 3). PRISm demonstrates preserved FEV_1_/FVC ratio (≥0.70) with reduced FEV_1_ (< 80% predicted value), with an estimated prevalence of 11% among adults in population-based studies (4). Both AL and PRISm have been independently associated with adverse health outcomes, including increased all-cause mortality and a heightened risk of CVD (5–8). Despite these substantial health implications, the biological mechanisms underlying the associations linking these distinct lung function impairment phenotypes to adverse health outcomes remain poorly understood (9–11).

Biological aging has recently emerged as a key driver of chronic disease onset and progression (12). Acting at the molecular, cellular, and systemic levels, it serves as a core mechanism underlying the development of various age-related conditions. Biological aging demonstrates strong links to detrimental clinical conditions, with manifestations including cancer, metabolic disorders, and CVD, which is a primary determinant of the heightened morbidity and mortality in patients with PRISm and AL (4, 13–17). To objectively quantify biological aging, researchers have developed a range of tools, of which phenotypic age and the frailty phenotype are the most commonly used (12, 18, 19). Phenotypic Age, calculated using chronological age combined with nine specific biomarkers, captures age-related changes at the molecular levels, providing an accurate assessment of biological aging. It has demonstrated strong predictive power for morbidity and mortality in population studies (20). In contrast, the frailty phenotype, introduced by Fried et al., highlights functional dimensions of aging, reflecting increased vulnerability resulting from age-related declines in physical, psychological, and social functioning. It is strongly associated with elevated risks of CVD, hospitalization, and mortality (21). Together, these two complementary measures provide a holistic understanding of aging by integrating molecular and functional perspectives.

Emerging evidence highlights a complex relationship between lung function impairment and biological aging. Lung function naturally declines with age, and certain chronic respiratory diseases, such as interstitial lung disease and idiopathic pulmonary fibrosis, are thought to be associated with accelerated aging processes (22). Among these, COPD has been strongly associated with biological aging markers. At the molecular level, COPD patients demonstrate shorter telomeres and accelerated telomere attrition, reflecting premature cellular senescence (23, 24). Epigenetic aging biomarkers, such as DNAmSkinBlood and DNAmGrimAge, provide further evidence supporting this association (25). Functionally, COPD is linked to a high prevalence of frailty, affecting up to 36% of patients (26). Longitudinal studies have confirmed the role of COPD in accelerating frailty progression. Notably, the PRISm phenotype, which is distinct from COPD, is associated with a more rapid progression of frailty, with an annual frailty index increase of 0.301 compared to 0.172 in COPD (27). Several critical knowledge gaps remain in the field. First, most studies on COPD are constrained by small sample sizes, cross-sectional designs, and short follow-up periods, resulting in insufficient longitudinal evidence. Moreover, the relationship between PRISm and biological aging markers also has not been systematically explored. Second, prior research has largely focused on isolated aging markers, lacking a comprehensive evaluation of both molecular and functional aging. Finally, the impact of biological aging on disease trajectory, clinical outcomes, and prognosis in patients with AL and PRISm remains poorly understood. Addressing these gaps is essential for identifying high-risk populations, informing targeted interventions, and advancing our understanding of aging in chronic lung diseases. Based on this background, using data from the UK Biobank, the present study aims to investigate whether biological aging mediates the association between lung function impairment and the risks of incident CVD and all-cause mortality. Furthermore, it evaluates the interaction and joint effects of biological aging and lung function on adverse health outcomes. We hypothesize that in individuals with impaired lung function, accelerated biological aging not only increases the risks of cardiovascular disease and all-cause mortality, but also acts as a key biological mechanism linking lung function impairment to adverse health outcomes.

## 2 Methods

### 2.1 Study population and design

The data for this study were derived from the UK Biobank, a large, population-based prospective cohort that enrolled approximately 500,000 individuals aged 40–69 years between 2006 and 2010 across 22 assessment centers in the UK. Participants have been followed for over 15 years through linkage to national health-related databases. At baseline, comprehensive data on demographics, lifestyle, and health status were collected via questionnaires, interviews, and biological samples (28). Informed consent was obtained from all participants, and ethical approval was granted by the North West Multi-Centre Research Ethics Committee. This study was approved by the UK Biobank (application number 203544), and all analyses were conducted in compliance with relevant privacy and ethical guidelines. The analysis excluded participants with: (1) invalid spirometry measurements; (2) incomplete covariate data; (3) insufficient data to calculate PhenoAgeAccel or frailty, or (4) pre-existing CVD at baseline. Additional details are provided in Supplementary Figure S1.

### 2.2 Assessments of spirometry

Participants underwent two to three pre-bronchodilator spirometry assessments during recruitment, and the highest FEV_1_ and FVC values from valid attempts were recorded. The predicted FEV_1_% was calculated using the ‘*RSpiro*’ package in R (version 4.4.0) based on Global Lung Function Initiative (GLI) 2012 reference equations. Baseline lung function phenotypes were classified into: (1) normal spirometry (FEV_1_/FVC ≥ 0.7 and FEV_1_ ≥ 80% of predicted), (2) PRISm (FEV_1_/FVC ≥ 0.7 and FEV_1_ < 80% of predicted), and (3) AL (FEV_1_/FVC < 0.7) (2, 7).

### 2.3 Assessment of biological aging

Phenotypic age and phenoAgeAccel were calculated based on previously established methodology, utilizing biomarkers from biological samples collected at recruitment (20). The phenotypic age-related biomarkers and their corresponding field IDs in the UK Biobank are detailed in Supplementary Table S1. The algorithms and corresponding R code are available in the ‘*BioAge*’ R package. PhenoAgeAccel was analyzed both as a continuous variable and stratified into quartiles. Frailty severity was assessed using the frailty phenotype, a widely validated assessment tool (21). This phenotype comprises five key components: weight loss, exhaustion, physical inactivity, slow walking speed, and weakness. Each component was adapted based on the available UK Biobank data (Supplementary Table S2). Frailty scores were determined by summing the components, with higher scores indicating greater frailty severity. Participants were classified into three groups: “normal” for those meeting none of the criteria, “pre-frail” for those meeting one or two criteria, and “frail” for those meeting three to five criteria. We employed two clinically accessible molecular and functional approaches to quantify biological aging from different dimensions, aiming to achieve robust and reliable results.

### 2.4 Outcome measurement and covariates

The main outcomes assessed in this study were all-cause mortality and CVD, while secondary outcomes included coronary artery disease (CAD), heart failure (HF), and ischemic stroke (IS). Diagnostic codes from the 10th edition of the International Classification of Diseases (ICD-10) were used to identify these conditions. Detailed code information used to define diseases is provided in Supplementary Table S3. The follow-up period spanned from the enrollment date to the earliest occurrence of a cardiovascular event, death, loss to follow-up, or the study’s endpoint (December 31, 2023).

Several potential confounders were considered covariates in this study. Age, gender (male/female), body mass index (BMI), Townsend deprivation index, smoking status (never/previous/current), drinking frequency (<3 times per week/≥3 times per week), and sleep status (lack/normal/excess) were collected through baseline questionnaires. Hyperlipidemia, diabetes, renal impairment, and hypertension (all classified as yes/no) were determined based on self-reports and diagnostic codes (Supplementary Table S3).

### 2.5 Statistical analysis

The baseline demographic and clinical characteristics were categorized and reported according to distinct lung function phenotypes. For variables following normal distributions, means with standard deviations were calculated. Non-normally distributed data were presented as medians with interquartile ranges (IQRs). Categorical data were described using frequencies and percentages. To compare continuous variables across groups, one-way ANOVA or the Kruskal–Wallis test was applied. The chi-square (*χ*^2^) test was used for comparisons of categorical variables.

Kaplan–Meier analysis was used to generate survival curves across lung function categories, and differences were evaluated using the log-rank test. Cox proportional hazards models were applied to examine the relationship between lung function phenotypes and adverse health outcomes. The Schoenfeld residuals method was employed to test the proportional hazards assumption, with no violations detected. Subgroup analyses by age (<60 versus ≥60 years) and gender (male versus female) were performed to evaluate effects across subpopulations. We incorporated interaction terms into the proportional hazards model to assess the potential interactions between lung function phenotypes and predefined stratification variables.

In addition, multiple linear regression models were applied to analyze the associations of lung function phenotypes with PhenoAgeAccel and frailty scores. Kaplan–Meier curves were constructed to visualize survival probabilities across different levels of biological aging. Cox models and restricted cubic splines (RCS) were applied to examine the independent relationships of PhenoAgeAccel and frailty scores with adverse health outcomes. Subsequently, causal mediation analysis was performed using the ‘*regmedint*’ package in R to assess the mediating effects of biological aging on survival data (29). Within the mediation framework, we decomposed the Total Effect (TE) of lung function impairment on adverse health outcomes into two components: the Natural Direct Effect (NDE), representing the effect independent of biological aging markers (PhenoAgeAccel and frailty scores), and the Natural Indirect Effect (NIE), capturing the effect operating through these aging markers. The proportion of the total effect mediated through the biological aging pathway was calculated as: proportion mediated = NIE/(NDE + NIE) × 100%.

Finally, we assessed the additive and multiplicative interactions between lung function impairment and biological aging. The assessment of multiplicative interaction effects was performed using likelihood ratio tests, which involved comparing models with and without interaction terms. For additive interactions, three metrics were computed: the Relative Excess Risk due to Interaction (RERI), the Attributable Proportion (AP), and the Synergy Index (SI) (30, 31). To investigate the joint effects, study participants were stratified into either 12 or 9 distinct groups based on three lung function phenotypes (normal, PRISm, and AL) combined with either PhenoAgeAccel quartiles or frailty phenotypes (normal, pre-frail, and frail). Hazard ratios for all-cause mortality and CVD incidence were calculated within these groups, with individuals having normal spirometry and either the lowest quartile of PhenoAgeAccel or a normal frailty phenotype serving as the reference group.

To ensure the robustness of the main findings, several sensitivity analyses were additionally conducted. First, both multiple imputation and random forest imputation were applied to handle missing values in the dataset. The relationships between lung function impairment and adverse outcomes, as well as the mediating role of biological aging, were then reassessed using the imputed complete dataset. Second, to minimize potential reverse causation bias, we excluded events that occurred during the first 2 years of follow-up. Third, considering the impact of the COVID-19 pandemic on global health, analyses were conducted by censoring data at December 31, 2019, marking the onset of the pandemic (32). Fourth, the mediation analysis of biological aging on the association between lung function impairment and CVD was reassessed after excluding participants with any pre-existing circulatory system disease (ICD-10: I00-I99). Finally, considering the potential impact of commonly used medications on lung function and aging biomarkers, we adjusted for the use of statins, β-blockers, and aspirin in the models and repeated the primary analyses.

All statistical analyses were conducted using R software (version 4.4.0). Statistical significance was defined as a two-sided *p-*value < 0.05.

## 3 Results

### 3.1 Baseline characteristic

Among the 502,132 participants in the UK Biobank, 349,456 individuals with the best spirometry measurements and complete covariates were included in the analysis. At baseline, 10.9% (38,257) of participants were diagnosed with PRISm, while 15.8% (55,152) were diagnosed with AL. The baseline characteristics of the study population, grouped by lung function phenotypes, are summarized in Table 1. Compared to the normal group, the AL group was more likely to be male and had a lower BMI, whereas the PRISm group exhibited a notably higher BMI. In addition, both the PRISm and AL groups showed a greater proportion of current smokers, generally poorer sleep quality, and a higher prevalence of comorbidities, including hypertension, diabetes, hyperlipidemia, and renal impairment. Both PRISm and AL groups exhibited accelerated biological aging, as indicated by elevated PhenoAgeAccel (0.96 and 0.78 vs. -0.33, respectively) and a higher proportion in the highest quartile of PhenoAgeAccel (32.7 and 31.8% vs. 22.3%). Frailty scores were also higher (0.79 and 0.63 vs. 0.53, respectively), with markedly higher proportions of pre-frail (45.5 and 39.6% vs. 37.4%, respectively) and frail individuals (6.4 and 4.3% vs. 2.4%, respectively). Participants excluded from the current analysis due to missing data were older, had a higher BMI, and experienced greater socioeconomic deprivation compared to those included in the analytical cohort (Supplementary Table S4). Moreover, individuals with cardiovascular disease at baseline exhibited poorer lung function (Supplementary Table S5).

**Table 1:** Baseline characteristics.

Characteristics	Normal (*N* = 256,047)	PRISm (*N* = 38,257)	AL (*N* = 55,152)	*p-*value
Age	55.96 (8.00)	56.38 (8.00)	59.11 (7.47)	<0.001
Male	113,775 (44.4%)	17,099 (44.7%)	30,796 (55.8%)	<0.001
BMI, kg/m^2^	27.25 (4.50)	29.16 (5.58)	26.68 (4.49)	<0.001
Townsend deprivation index	−1.66 (2.86)	−1.19 (3.12)	−1.18 (3.15)	<0.001
Drinking frequency:				<0.001
<3 times per week	137,091 (53.5%)	22,856 (59.7%)	28,241 (51.2%)	
≥3 times per week	118,956 (46.5%)	15,401 (40.3%)	26,911 (48.8%)	
Smoking status				<0.001
Never	145,333 (56.8%)	19,604 (51.2%)	22,540 (40.9%)	
Previous	90,415 (35.3%)	13,938 (36.4%)	22,085 (40.0%)	
Current	20,299 (7.9%)	4,715 (12.3%)	10,527 (19.1%)	
Sleep status				<0.001
Normal	192,916 (75.3%)	27,476 (71.8%)	40,653 (73.7%)	
Lack	59,676 (23.3%)	9,894 (25.9%)	13,388 (24.3%)	
Excess	3,455 (1.3%)	887 (2.3%)	1,111 (2.0%)	
Hypertension	60,701 (23.7%)	12,755 (33.3%)	16,001 (29.0%)	<0.001
Diabetes	9,440 (3.7%)	3,316 (8.7%)	2,677 (4.9%)	<0.001
Hyperlipidaemia	33,769 (13.2%)	7,074 (18.5%)	9,269 (16.8%)	<0.001
Renal impairment	3,249 (1.3%)	823 (2.2%)	958 (1.7%)	<0.001
FEV_1_, L	3.05 (0.72)	2.20 (0.53)	2.41 (0.77)	<0.001
FVC, L	3.92 (0.93)	2.90 (0.70)	3.75 (1.10)	<0.001
FEV_1_/FVC	0.78 (0.04)	0.76 (0.04)	0.64 (0.07)	<0.001
FEV_1_%	99.18 (11.93)	71.98 (7.71)	77.64 (18.07)	<0.001
FVC%	100.55 (12.08)	74.81 (8.59)	94.34 (18.81)	<0.001
PhenoAgeAccel	−0.33 (4.30)	0.96 (5.19)	0.78 (4.80)	<0.001
PhenoAgeAccel (quartile)				<0.001
Q1	56,765 (26.8%)	6,361 (20.2%)	9,312 (20.3%)	
Q2	55,060 (26.0%)	6,973 (22.2%)	10,444 (22.7%)	
Q3	52,971 (25.0%)	7,823 (24.9%)	11,569 (25.2%)	
Q4	47,183 (22.3%)	10,292 (32.7%)	14,606 (31.8%)	
Serum glucose, mmol/L	5.07 (1.08)	5.27 (1.56)	5.10 (1.15)	<0.001
Mean cell volume, fL	82.70 (5.12)	82.53 (5.34)	83.64 (5.50)	<0.001
White blood cell count, 10^9/L	6.74 (2.13)	7.21 (2.02)	7.12 (2.11)	<0.001
Lymphocyte, %	29.07 (7.25)	28.57 (7.32)	27.93 (7.39)	<0.001
Red cell distribution width, %	13.42 (0.91)	13.54 (1.01)	13.53 (0.96)	<0.001
Creatine, μmol/L	72.06 (16.30)	72.09 (21.83)	73.88 (17.09)	<0.001
Median CRP (IQR), mg/dL	0.12 (0.06, 0.24)	0.19 (0.09, 0.38)	0.14 (0.07, 0.29)	<0.001
Alkaline phosphatase, U/L	81.95 (25.20)	86.81 (27.08)	84.64 (27.30)	<0.001
Albumin, g/L	45.41 (2.57)	44.97 (2.65)	44.99 (2.62)	<0.001
Frailty scores	0.53 (0.76)	0.79 (0.96)	0.63 (0.87)	<0.001
Frailty phenotype				<0.001
Normal	147,553 (60.2%)	17,344 (48.2%)	29,294 (56.2%)	
Pre-frail	916,44 (37.4%)	16,376 (45.5%)	20,635 (39.6%)	
Frail	5,934 (2.4%)	2,293 (6.4%)	2,221 (4.3%)	
Weight loss	38,390 (15.2%)	5,440 (14.5%)	8,017 (14.8%)	<0.001
Tiredness	26,231 (10.5%)	5,497 (14.8%)	5,972 (11.2%)	<0.001
Slow walking speed	13,124 (5.1%)	4,578 (12.1%)	5,189 (9.5%)	<0.001
Weakness	41,040 (16.0%)	10,399 (27.2%)	10,981 (19.9%)	<0.001
Physical inactivity	17,142 (6.7%)	4,548 (12.0%)	5,215 (9.5%)	<0.001

Data are reported as mean (SD) or *n* (%), unless stated otherwise.

PRISm, preserved ratio impaired spirometry; AL, airflow limitation.

### 3.2 Associations of lung function phenotypes with mortality and CVD incidence

The associations between baseline lung function phenotypes and adverse health outcomes are shown in Supplementary Figures S2A,B and Table 2. After multivariate adjustment, both PRISm and AL demonstrated significant associations with increased risks of adverse outcomes. Compared with participants with normal lung function, the PRISm group had a higher risk of all-cause mortality (HR: 1.39, 95%CI: 1.34–1.44), CVD (HR: 1.27, 95%CI: 1.23–1.31), IS (HR: 1.31, 95%CI: 1.21–1.41), CAD (HR: 1.19, 95%CI: 1.15–1.24), and HF (HR: 1.67, 95%CI: 1.58–1.77). Similarly, AL was associated with increased risks of all-cause mortality (HR: 1.49, 95%CI: 1.45–1.53), CVD (HR: 1.22, 95%CI: 1.19–1.25), IS (HR: 1.21, 95%CI: 1.13–1.29), CAD (HR: 1.15, 95%CI: 1.11–1.18), and HF (HR: 1.65, 95%CI: 1.57–1.74). Age-and gender-stratified analyses are presented in Supplementary Figure S3, showing significant interactions for all outcomes (all *P* for interaction < 0.05).

**Table 2:** Hazard ratios (95%CI) of lung function phenotypes with all-cause mortality and CVD outcomes.

	Normal	PRISm	AL
All-cause mortality			
No. of events/person-years	16,230/372,5,136	4,093/54,5,708	7,736/77,6,816
Model 1^a^	1.00 (Reference)	1.49 (1.44–1.54)	1.54 (1.50–1.59)
Model 2^b^	1.00 (Reference)	1.39 (1.34–1.44)	1.49 (1.45–1.53)
CVD			
No. of events/person-years	22,650/3,409,940	4,709/46,9,253	7,504/672,752
Model 1	1.00 (Reference)	1.33 (1.29–1.37)	1.25 (1.21–1.28)
Model 2	1.00 (Reference)	1.27 (1.23–1.31)	1.22 (1.19–1.25)
IS			
No. of events/person-years	3,963/3,529,139	835/492,967	1380/708,848
Model 1	1.00 (Reference)	1.38 (1.28–1.48)	1.24 (1.16–1.31)
Model 2	1.00 (Reference)	1.31 (1.21–1.41)	1.21 (1.13–1.29)
CAD			
No. of events/person-years	17,229/3,438,759	3,382/476,262	5,380/683,442
Model 1	1.00 (Reference)	1.25 (1.21–1.30)	1.17 (1.13–1.21)
Model 2	1.00 (Reference)	1.19 (1.15–1.24)	1.15 (1.11–1.18)
HF			
No. of events/person-years	5,094/3,528,856	1,551/490,695	2413/706,509
Model 1	1.00 (Reference)	1.79 (1.69–1.90)	1.70 (1.62–1.79)
Model 2	1.00 (Reference)	1.67 (1.58–1.77)	1.65 (1.57–1.74)

^a^Adjusted for age, gender, BMI, smoking status (never/former/current).

^b^Adjusted for sleep status (normal/excess/lack), deprivation index, drinking frequency (<3 or ≥3 times a week), hypertension (yes/no), diabetes (yes/no), hyperlipidemia (yes/no), and renal impairment (yes/no) based on model 1.

CI, confidence interval; PRISm, preserved ratio impaired spirometry; AL, airflow limitation; CVD, cardiovascular disease; IS, ischemic Stroke; CAD, coronary artery disease; HF, heart failure.

### 3.3 Association of lung function phenotypes with biological aging

In multivariable-adjusted analyses, both impaired lung function phenotypes showed positive associations with PhenoAgeAccel (PRISm: *β* = 0.655, 95%CI: 0.604–0.705; AL: *β* = 0.774, 95%CI: 0.731–0.818) and frailty score (PRISm: *β* = 0.129, 95%CI: 0.120–0.137; AL: *β* = 0.085, 95%CI: 0.077–0.092) (Supplementary Table S6).

### 3.4 Association of biological aging with mortality and CVD incidence

As shown in Supplementary Figure S2, individuals with the highest quartile (Q4) of PhenoAgeAccel and those classified as frail exhibited the greatest risks of mortality (C and E) and CVD incidence (D and F). In multivariable-adjusted models, each 1-year increase in PhenoAgeAccel was associated with elevated risks of all-cause mortality (HR: 1.06, 95%CI: 1.06–1.06), CVD (HR: 1.03, 95%CI: 1.03–1.04), IS (HR: 1.04, 95% CI: 1.03–1.04), CAD (HR: 1.03, 95%CI: 1.02–1.03), and HF (HR: 1.06, 95%CI: 1.05–1.06) (all *p* < 0.001, Supplementary Table S7). Similar findings were observed when PhenoAgeAccel was analyzed as a continuous variable, using the median of each quantile (all *P* trend < 0.001). A non-linear association was found between PhenoAgeAccel and both mortality and CVD incidence (both *P* for non-linearity < 0.001, Supplementary Figure S4).

Similarly, each 1-point increase in frailty scores was associated with elevated risks of all-cause mortality (HR: 1.27, 95%CI: 1.25–1.28), CVD (HR: 1.19, 95%CI: 1.18–1.21), IS (HR: 1.14, 95%CI: 1.11–1.18), CAD (HR: 1.18, 95%CI: 1.17–1.20), and HF (HR: 1.30, 95%CI: 1.27–1.33) (all *p* < 0.001; Supplementary Table S7). A significant dose–response association was observed between frailty phenotype and adverse outcomes (all *P* trend < 0.001). The association was linear for CVD incidence (*P* for non-linearity = 0.150) but non-linear for all-cause mortality (*P* for non-linearity < 0.001, Supplementary Figure S4).

### 3.5 Mediation analysis

**Figure 1:**
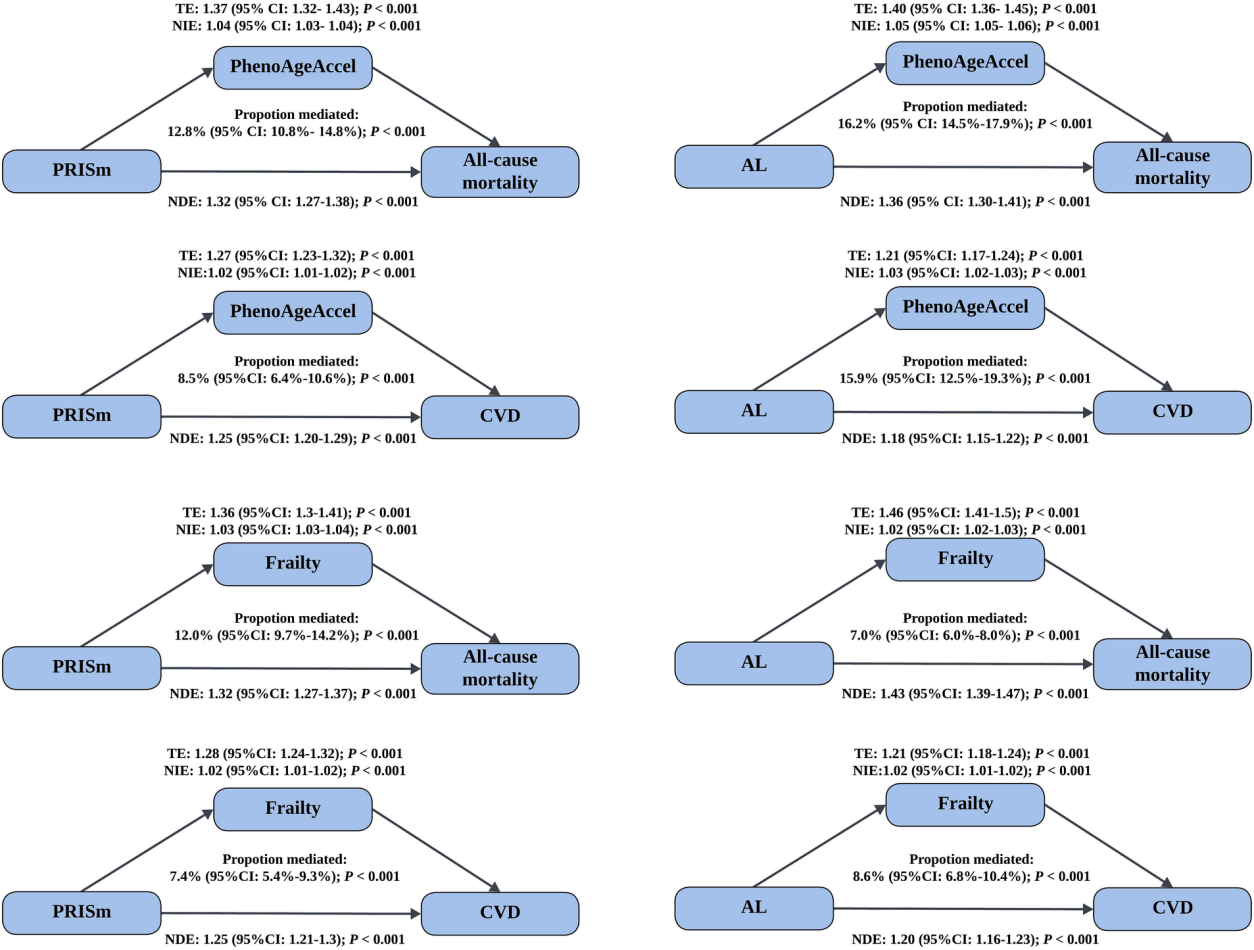
Mediated effect of biological aging on the association of lung function impairment with mortality and CVD incidence. Models were adjusted for age, gender, BMI, smoking status (never/former/current), sleep status (normal/excess/lack), deprivation index, drinking frequency (<3 or ≥3 times a week), hypertension (yes/no), diabetes (yes/no), hyperlipidemia (yes/no), and renal impairment (yes/no). CI, confidence interval; PhenoAgeAccel, phenotypic age acceleration; CVD, cardiovascular disease; TE, total effect; NIE, natural indirect effect; NDE, natural direct effect.

Mediation analyses demonstrated that biological aging partially mediated the association between lung function impairment and adverse health outcomes (Figure 1). For PRISm, PhenoAgeAccel mediated 12.8% (95%CI: 10.8–14.8%) of its association with all-cause mortality and 8.5% (95%CI: 6.4–10.6%) with CVD incidence. The mediation effects were more pronounced for AL, with PhenoAgeAccel mediating 16.2% (95%CI: 14.5–17.9%) and 15.9% (95% CI: 12.5–19.3%) of its associations with all-cause mortality and CVD incidence, respectively (all *p* < 0.001). Further analyses assessed the mediation proportion of nine phenotypic age-related biomarkers (Supplementary Tables S8–S11). Among them, ln-CRP exhibited the strongest mediation effect, ranging from 7.1% (95%CI: 6.1–7.1%) to 10.8% (95%CI: 8.6–13.1%). Using frailty as an alternative indicator of biological aging, similar mediation effects were observed: frailty mediated 12.0% (95%CI: 9.7–14.2%) and 7.4% (95%CI: 5.4–9.3%) of PRISm’s associations with all-cause mortality and CVD incidence, respectively, and 7.0% (95%CI: 6.0–8.0%) and 8.6% (95%CI: 6.8–10.4%) of the corresponding associations for AL (all *p* < 0.001). Among the five frailty components, slow walking speed had the largest mediation contribution in most analyses, with proportions ranging from 2.6% (95%CI: 1.7–3.5%) to 5.8% (95%CI: 4.4–7.1%) (Supplementary Tables S12, S13). In analyses of individual CVD components, the mediation proportions of two biological aging indicators ranged from 3.9% (95%CI: 0.4–7.4%) to 20.7% (95%CI: 13.0–28.3%) (all *p* < 0.05, Table 3).

**Table 3:** The mediating effect of biological aging on the association between lung function phenotypes and the three individual CVD components.

Mediator		HR (95%CI)			% mediated	*p*-value
		Total effect	Natural direct effect	Natural indirect effect		
PhenoAgeAccel	HF					
	PRISm	1.67 (1.57–1.79)	1.62 (1.52–1.73)	1.03 (1.02–1.03)	6.9% (5.4–8.4%)	<0.001
	AL	1.66 (1.57–1.76)	1.60 (1.51–1.69)	1.04 (1.04–1.05)	10.6% (8.7–12.4%)	<0.001
	IS					
	PRISm	1.27 (1.17–1.39)	1.25 (1.15–1.36)	1.02 (1.02–1.03)	10.4% (5.4–15.4%)	<0.001
	AL	1.19 (1.11–1.27)	1.16 (1.08–1.24)	1.03 (1.02–1.03)	16.4% (7.5–25.3%)	<0.001
	CAD					
	PRISm	1.21 (1.16–1.26)	1.19 (1.14–1.24)	1.01 (1.01–1.02)	7.7% (4.7–10.6%)	<0.001
	AL	1.13 (1.09–1.17)	1.11 (1.07–1.15)	1.02 (1.02–1.03)	20.7% (13.0–28.3%)	<0.001
Frailty	HF					
	PRISm	1.70 (1.59–1.81)	1.65 (1.55–1.76)	1.02 (1.02–1.03)	5.8% (4.2–7.4%)	<0.001
	AL	1.62 (1.54–1.71)	1.60 (1.51–1.68)	1.02 (1.02–1.02)	5.5% (4.4–6.6%)	<0.001
	IS					
	PRISm	1.34 (1.24–1.45)	1.32 (1.22–1.43)	1.01 (1.00–1.02)	3.9% (0.4–7.4%)	0.026
	AL	1.22 (1.14–1.30)	1.21 (1.13–1.29)	1.01 (1.01–1.01)	5.5% (2.3–8.7%)	<0.001
	CAD					
	PRISm	1.21 (1.16–1.25)	1.18 (1.14–1.23)	1.01 (1.01–1.02)	8.8% (5.6–12.1%)	<0.001
	AL	1.13 (1.09–1.17)	1.12 (1.08–1.15)	1.01 (1.01–1.02)	12.9% (8.7–17.1%)	<0.001

HR, hazard ratio; CI, confidence interval; HF, heart Failure; IS, ischemic Stroke; CAD, coronary artery disease; PRISm, preserved ratio impaired spirometry; AL, airflow limitation.

### 3.6 Interaction and joint analysis

Significant additive and multiplicative interactions were observed between lung function phenotypes and biological aging for all-cause mortality, except for the non-significant multiplicative interaction between PRISm and frailty (Table 4). For CVD incidence, additive interactions were identified between both PRISm and AL with PhenoAgeAccel, whereas AL also showed a significant multiplicative interaction with frailty (Table 4). Biological aging was independently associated with increased risks of all-cause mortality and CVD incidence across lung function phenotypes, with stronger associations observed for mortality in individuals with impaired lung function (Supplementary Tables S14, S15). Among participants with normal lung function, the highest quartile (Q4) of PhenoAgeAccel was associated with an increased mortality risk (HR: 1.68, 95%CI: 1.59–1.76), which was further elevated in PRISm (HR: 1.85, 95%CI: 1.65–2.07) and AL patients (HR: 2.08, 95%CI: 1.91–2.26). Similarly, frailty demonstrated progressively stronger associations with mortality, ranging from normal lung function (HR: 1.90, 95%CI: 1.76–2.05) to PRISm (HR: 2.19, 95%CI: 1.96–2.45) and AL (HR: 2.35, 95%CI: 2.15–2.56) (Supplementary Tables S14, S15).

**Table 4:** Interaction effects of lung function phenotypes and biological aging on all-cause mortality and CVD incidence.

	Multiplicative interaction		Additive interaction		
	HR (95%CI)	*p-*value	RERI (95%CI)	AP (95%CI)	SI (95%CI)
All-cause mortality					
PRISm and PhenoAgeAccel	1.10 (1.02–1.18)	0.011	0.45 (0.30–0.59)	0.18 (0.13–0.23)	1.42 (1.28–1.58)
PRISm and Frailty	1.01 (0.91–1.12)	0.818	0.41 (0.14–0.67)	0.14 (0.06–0.22)	1.28 (1.10–1.49)
AL and PhenoAgeAccel	1.15 (1.08–1.22)	<0.001	0.60 (0.48–0.72)	0.22 (0.19–0.26)	1.55 (1.43–1.69)
AL and Frailty	1.14 (1.04–1.25)	0.007	1.02 (0.72–1.32)	0.28 (0.22–0.35)	1.64 (1.45–1.86)
CVD					
PRISm and PhenoAgeAccel	1.03 (0.96–1.10)	0.466	0.14 (0.03–0.24)	0.08 (0.02–0.13)	1.22 (1.05–1.42)
PRISm and Frailty	0.92 (0.82–1.03)	0.156	0.02 (−0.2–0.24)	0.01 (−0.09–0.12)	1.02 (0.83–1.26)
AL and PhenoAgeAccel	1.05 (0.99–1.11)	0.120	0.14 (0.06–0.22)	0.08 (0.04–0.13)	1.26 (1.10–1.45)
AL and Frailty	1.12 (1.00–1.25)	0.048	1.05 (0.69–1.42)	0.28 (0.21–0.36)	1.64 (1.42–1.88)

All models adjusted for age, gender, BMI, smoking status (never/former/current), sleep status (normal/excess/lack), deprivation index, drinking frequency (<3 or ≥3 times a week), hypertension (yes/no), diabetes (yes/no), hyperlipidemia (yes/no), and renal impairment (yes/no).

CI, confidence interval; CVD, cardiovascular disease; HR, hazard ratio; RERI, relative excess risk; AP, attributable proportion; SI, synergy index; PRISm, preserved ratio impaired spirometry; AL, airflow limitation.

**Figure 2:**
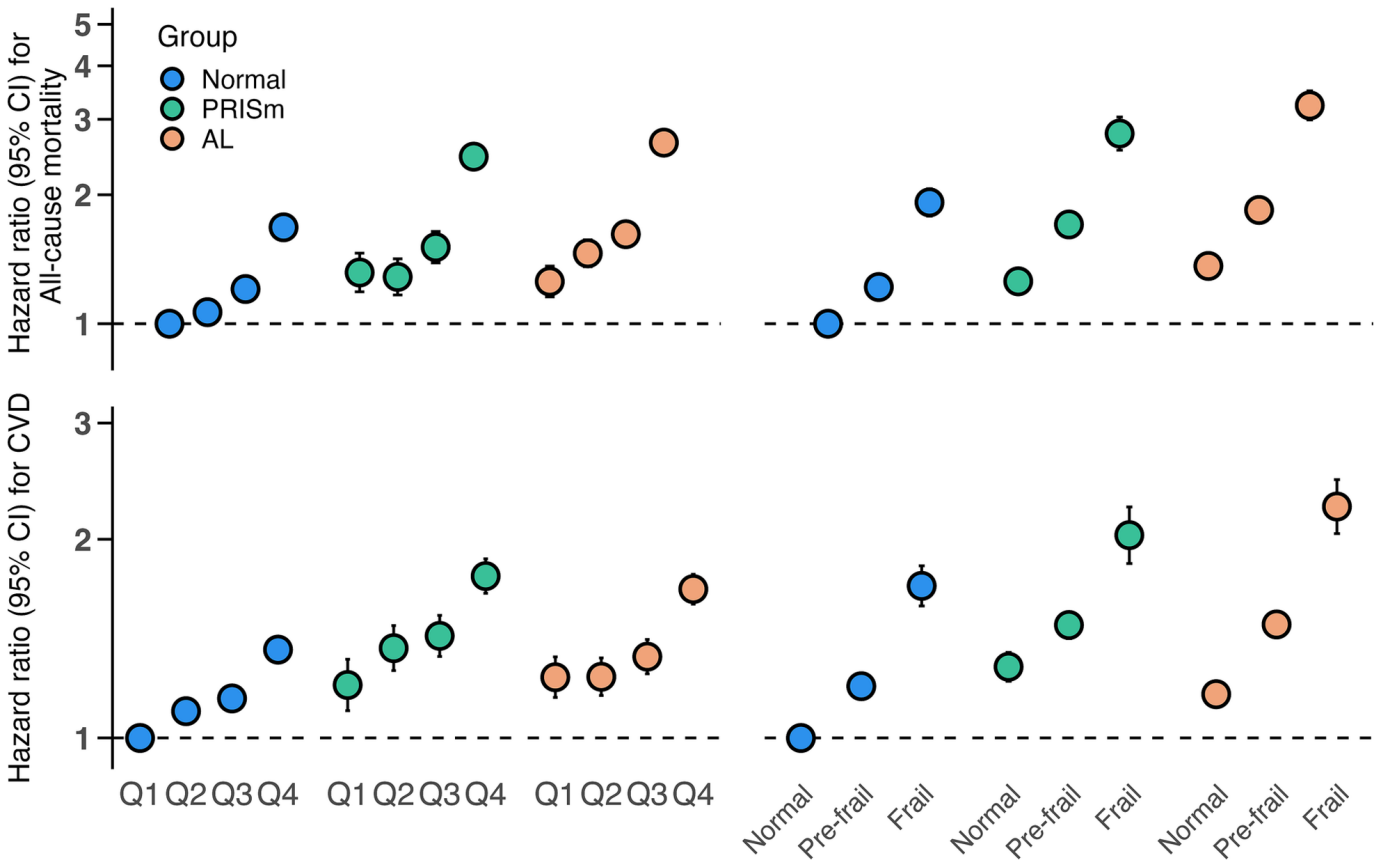
Joint associations of biological aging (PhenoAgeAccel and frailty phenotype) and lung function phenotypes with mortality and CVD incidence. Models were adjusted for age, gender, BMI, smoking status (never/former/current), sleep status (normal/excess/lack), deprivation index, drinking frequency (<3 or ≥3 times a week), hypertension (yes/no), diabetes (yes/no), hyperlipidemia (yes/no), and renal impairment (yes/no). CI, confidence interval; PhenoAgeAccel, phenotypic age acceleration; CVD, cardiovascular disease.

Figure 2 illustrates the joint effects of lung function impairment and biological aging on adverse health outcomes. Compared with participants with normal lung function and the lowest quartile (Q1) of PhenoAgeAccel, those with PRISm or AL combined with the highest quartile (Q4) demonstrated significantly increased risks of all-cause mortality (PRISm: HR: 2.46, 95%CI: 2.30–2.62; AL: HR: 2.65, 95%CI: 2.51–2.80) and CVD incidence (PRISm: HR: 1.76, 95%CI: 1.66–1.87; AL: HR: 1.68, 95%CI: 1.60–1.77) (Supplementary Table S16). Similar patterns were observed when frailty was used as an alternative aging marker, with higher risks in frail individuals with PRISm (mortality: HR: 2.78, 95%CI: 2.54–3.04; CVD: HR: 2.03, 95%CI: 1.84–2.24) and AL (mortality: HR: 3.23, 95%CI: 2.99–3.50; CVD: HR: 2.24, 95%CI: 2.04–2.46) (Supplementary Table S17), compared to non-frail individuals with normal lung function.

### 3.7 Sensitive analysis

The results remained robust after addressing missing covariates through multiple imputation and random forest imputation (Supplementary Tables S18–S21), excluding participants with follow-up durations of less than 2 years (Supplementary Tables S22, 23), setting the study endpoint to December 31, 2019 (Supplementary Tables S24, 25), excluding participants with any circulatory system disease (ICD-10: I00-I99) at baseline (Supplementary Tables S26, 27), or adjusting for the use of common medications, including statins, β-blockers, and aspirin, as covariates in the model (Supplementary Tables S28, S29).

## 4 Discussion

This large prospective cohort study provides several key findings: (1) biological aging mediates the association between lung function impairment and the risks of mortality and CVD; (2) significant multiplicative and additive interactions are present between biological aging and lung function impairment in relation to mortality; and (3) individuals with both impaired lung function and accelerated biological aging exhibit the highest risks of mortality and CVD.

These findings contribute to a more comprehensive understanding of the association between lung function impairment and adverse health outcomes. Previous studies by Wijnant et al. (3) reported higher all-cause mortality and cardiovascular mortality rates in individuals with PRISm, while Zheng et al. (7) found that both PRISm and AL were independently associated with cardiovascular outcomes. In addition, findings from a large-scale Danish cohort study further substantiated these associations (33). Building on this evidence, our study extends the understanding by investigating the potential biological mechanisms involved, particularly highlighting the mediating role of biological aging in the pathway from lung function impairment to adverse outcomes. These insights may facilitate the development of targeted interventions.

Our findings suggest that lung function impairment accelerates biological aging, with PRISm having a more pronounced effect than AL. This finding extends previous research, which has predominantly focused on aging processes associated with COPD. For instance, Savale et al. (61) reported shortened telomeres in individuals with COPD, and the BONE cohort study confirmed accelerated telomere attrition in this population (23, 24). Additionally, Da Silva et al. (34) demonstrated enhanced immune aging in COPD patients. Regarding functional aging, although COPD patients are twice as likely to develop frailty compared to the general population, our findings suggest that PRISm may have an even greater impact on frailty, consistent with results from the English Longitudinal Study of Aging (27, 35, 36). We also observed significant associations of both PhenoAgeAccel and frailty with increased risks of cardiovascular disease (CVD) and all-cause mortality, which aligns with previous research (21, 37). Based on these observations, a mediation analysis was conducted. Specifically, we found that PhenoAgeAccel mediated 8.5–16.2% of the total effects of PRISm and AL on all-cause mortality and cardiovascular disease (CVD) incidence, with mediation effects for individual CVD components ranging from 6.9 to 20.7%. This mediation effect suggests that biological aging, as captured by PhenoAgeAccel, plays a crucial role in linking lung function impairment to adverse health outcomes. Several underlying mechanisms may account for the association between lung function impairment and PhenoAgeAccel. Smoking and environmental pollutants, such as PM_2.5_ and NO_2_, which are major risk factors for PRISm and AL, have been strongly associated with accelerated aging, as indicated by PhenoAgeAccel (18, 38). These effects are likely mediated by chronic low-grade inflammation. Both smoking and air pollution can trigger systemic inflammation, and the polygenic risk score for PhenoAge is closely related to inflammatory markers (39–41). This suggests that chronic inflammation may serve as a key pathway linking these risk factors to accelerated biological aging as captured by PhenoAgeAccel. In our component-specific mediation analyses, C-reactive protein (CRP) demonstrated the highest mediation proportion among all PhenoAgeAccel components, highlighting inflammation as a key pathway. Additionally, immune dysregulation may play a crucial role in this process. Blood biomarkers associated with PhenoAgeAccel, such as lymphocyte percentage, red cell distribution width, and mean corpuscular volume, reflect changes in the immune system. Moreover, genes related to phenotypic age have been found to be overexpressed in immune-related pathways (41). Research indicates that individuals with COPD often exhibit immune dysfunction, characterized by a significant imbalance in T cell subsets, enhanced Th17 response and elevated pro-inflammatory cytokines (42–46). Immune dysregulation is considered a central mechanism in the progression of COPD. Taken together, these findings suggest that impaired lung function accelerates biological aging through the initiation of chronic low-grade inflammation and immune dysregulation, thereby increasing the risk of CVD and mortality.

Frailty exhibited significant, though relatively moderate, mediating effects between lung function impairment and mortality/CVD. It accounted for 7.0–12.0% of the relationship between lung function impairment and mortality/CVD incidence, and 3.9–12.9% for individual CVD components. Lung function impairment may promote the development of frailty through several mechanisms. First, PRISm, COPD, and frailty share several common risk factors, such as aging, systemic inflammation, and tobacco exposure, suggesting the existence of shared biological pathways that trigger a cascade of physiological deterioration, affecting both lung function and overall physical reserve capacity (4, 5, 33, 35, 47). Second, sarcopenia may be an important bridge linking lung function impairment to frailty (48–51). COPD and PRISm patients often experience systemic hypoxia, which induces a shift in skeletal muscle composition from type I oxidative fibers to type II glycolytic fibers, resulting in decreased muscle endurance (52). Dyspnea-related physical inactivity further contributes to muscle atrophy due to disuse. Additionally, the combination of hypoxia and chronic inflammation (e.g., elevated IL-1, IL-6 and TNF-α levels) promotes protein degradation, inhibits protein synthesis, and accelerates the decline in muscle mass and function, ultimately driving the development of frailty (53, 54). Notably, among all frailty components, slow walking speed exhibited the strongest mediating effect, suggesting that lower limb dysfunction may be a key pathway linking lung function impairment to adverse outcomes. This highlights the importance of intervention strategies to improve mobility in individuals with impaired lung function.

In this analysis, we observed a robust interaction effect between lung function phenotypes and biological aging markers. Specifically, the association between biological aging and mortality risk was more pronounced among individuals with impaired lung function. These findings underscore the urgency of prioritizing aging-targeted interventions, including dietary habits, lifestyle modifications, and pharmacological treatments, especially for individuals with irreversible lung function impairment (55–59).

However, several limitations should be acknowledged. First, the use of pre-bronchodilator spirometry may have affected classification accuracy, particularly for AL. Incorporating post-bronchodilator tests in future studies could improve phenotype characterization and enhance result validity. Second, lung function was measured only once, which may have led to misclassification, as participants could shift between phenotypes during follow-up (7). Third, biomarkers and frailty phenotypes were assessed only at baseline, limiting longitudinal interpretation. Repeated measurements could better capture temporal changes and their implications. Fourth, individuals with baseline CVD were excluded to strengthen causal inference. However, this may have also excluded high-risk individuals with severe lung impairment and subclinical cardiovascular abnormalities, potentially underestimating the association between lung function, accelerated aging, and CVD. Finally, the generalizability of our findings is limited by the characteristics of the UK Biobank cohort, which mainly includes middle-aged and older adults of European ancestry with higher health awareness and healthier lifestyles (60). Therefore, future studies in more diverse populations are warranted to validate and extend these findings.

## 5 Conclusion

Based on this large-scale prospective cohort study, our findings advance our understanding of how biological aging mechanisms mediate the relationship between lung function impairment and adverse health outcomes. The synergistic interaction between lung function impairment and biological aging highlights the need for comprehensive assessments and targeted interventions. Future research should prioritize the identification of susceptible populations and the development of therapeutic interventions to mitigate accelerated biological aging, particularly in patients with irreversible lung function impairment. These findings provide a foundation for personalized preventive strategies and novel therapies targeting aging-related pathways.

## Data Availability

Publicly available datasets were analyzed in this study. This data can be found: https://www.ukbiobank.ac.uk/.
