# Supplementary material for: Associations of lung function impairment and biological aging with mortality and cardiovascular disease incidence: findings from UK biobank participants
Source: Front Public Health. 2025 Jul 10;13:1635195. doi: 10.3389/fpubh.2025.1635195 (PMC12287072; doi:10.3389/fpubh.2025.1635195)
Supplement: Supplementary file 1 [file Table_1.docx]

**Associations of lung function impairment and biological aging with mortality and cardiovascular disease incidence: A prospective cohort study**

**Supplementary materials:**

**Table S1** Phenotypic age-related biomarkers and corresponding UK Biobank data fields

**Table S2** Definition of frailty phenotype

**Table S3** Disease definitions (ICD-10) used in the UK biobank study

**Table S4** Baseline characteristics of participants included or excluded from analyses due to missing information

**Table S5** Baseline characteristics of participants based on pre-existing CVD status

**Table S6** Association between lung function phenotypes and biological aging

**Table S7** Associations of biological aging with mortality and CVD incidence

**Table S8** Association of PRISm with all-cause mortality mediated by 9 phenotypic age-related biomarkers

**Table S9** Association of airflow limitation with all-cause mortality mediated by 9 phenotypic age-related biomarkers

**Table S10** Association of PRISm with CVD incidence mediated by 9 phenotypic age-related biomarkers

**Table S11** Association of airflow limitation with CVD incidence mediated by 9 phenotypic age-related biomarkers

**Table S12** Association of PRISm with all-cause mortality and CVD incidence mediated by each frailty component

**Table S13** Association of airflow limitation with all-cause mortality and CVD incidence mediated by each frailty component

**Table S14** Associations of PhenoAgeAccel with mortality and CVD incidence by lung function phenotypes

**Table S15** Associations of frailty with mortality and incident CVD incidence by lung function phenotypes

**Table S16** The joint association of lung function phenotypes and PhnoAgeAccel quartiles with the risk of mortality and CVD incidence

**Table S17** The joint association of lung function phenotypes and frailty status with the risk of mortality and CVD incidence

**Table S18** Hazard ratios (95%CI) of lung function phenotypes for all-cause mortality and CVD incidence by multiple imputation

**Table S19** The mediating effect of biological aging on the association between lung function phenotypes and primary outcomes by multiple imputation

**Table S20** Hazard ratios (95%CI) of lung function phenotypes for all-cause mortality and CVD incidence by random forest imputation

**Table S21** The mediating effect of biological aging on the association between lung function phenotypes and primary outcomes by random forest imputation

**Table S22** Hazard ratios (95%CI) of lung function phenotypes for all-cause mortality and CVD incidence, excluding events in the first 2 years

**Table S23** The mediating effect of biological aging on the association between lung function phenotypes and primary outcomes , excluding events in the first 2 years

**Table S24** Hazard ratios (95%CI) of lung function phenotypes for all-cause mortality and CVD incidence before COVID-2019

**Table S25** The mediating effect of biological aging on the association between lung function phenotypes and primary outcomes before COVID-2019

**Table S26** Hazard ratios (95%CI) of lung function phenotypes for CVD incidence excluding patients with any cardiovascular disease at baseline

**Table S27** The mediating effect of biological aging on the association between lung function phenotypes and CVD incidence,excluding patients with any cardiovascular disease at baseline

**Table S28** Hazard ratios (95%CI) of lung function phenotypes for all-cause mortality and CVD incidence, incorporating the use of specific medications

**Table S29** The mediating effect of biological aging on the association between lung function phenotypes and primary outcomes, incorporating the use of specific medications

**Fig. S1** The flow chart of the selection of the study population

**Fig. S2** Kaplan-Meier survival and CVD-free survival curves stratified by lung function phenotypes, PhenoAgeAccel, and frailty phenotype

**Fig. S3** Hazard ratios (HRs) for mortality/CVD incidence and lung function phenotypes

**Fig. S4** Restricted cubic splines (RCS) for associations of accelerated biological aging with all-cause mortality and CVD incidence

**Table S1** Phenotypic age-related biomarkers and corresponding UK Biobank data fields

| **Labels in the current study** | **Full name in UK Biobank data dictionary** | **Field ID** |
| --- | --- | --- |
| Lymphocyte (%) | Lymphocyte percentage | 30180 |
| Mean cell volume (fL) | Mean sphered cell volume | 30270 |
| Serum glucose (mg/dL) | Glucose | 30740 |
| Red cell distribution width (%) | Red blood cell (erythrocyte) distribution width | 30070 |
| White blood cell count (1000 cells/μL) | White blood cell (leukocyte) count | 30000 |
| Albumin (g/dL) | Albumin | 30600 |
| Creatinine (mg/dL) | Creatinine | 30700 |
| C-reactive protein (mg/dL) | C-reactive protein | 30710 |
| Alkaline phosphatase (U/L) | Alkaline phosphatase | 30610 |

**Table S2** Definition of frailty phenotype

| **Frailty components** | **UK Biobank question** |
| --- | --- |
| Weight loss | Question: Compared with one year ago, has your weight changed?  Yes - lost weight=1;  No - weigh about the same/Yes - gained weight=0 |
| Exhaustion | Question: Over the past two weeks, how often have you felt tired or had little energy?  Not at all/Several days=0;  More than half the days/Nearly every day=1Yes - lost weight=1; |
| Slow walking speed | Question: How would you describe your usual walking pace?  Slow pace=1;  Steady average pace/Brisk pace=0 |
| Weakness | Grip strength was assessed isometrically using a calibrated J00105 hydraulic hand dynamometer (Lafayette Instrument Company, IN, USA), separately in the left and right arms. The average of the right and left measurement was used here, or the available one when either measurement is missing1. Cut-offs used to define low grip strength:  Males:  BMI ≤ 24 & grip strength ≤ 29;  24.1 ≤ BMI ≤ 28 & grip strength ≤ 30;  BMI > 28 & grip strength ≤ 32  Females:  BMI ≤ 23 & grip strength ≤ 17;  23.1 ≤ BMI ≤ 26 & grip strength ≤ 17.3;  26.1 ≤ BMI ≤ 29 & grip strength ≤ 18;  BMI > 29 & grip strength ≤ 21 |
| Physical inactivity | Question: In the last 4 weeks did you spend any time doing the following?  Walking for pleasure = 0;  Strenuous sports = 0;  Light DIY (eg: pruning, watering the lawn):  Frequency of once per week or less = 1;  Frequency of more than once per week = 0;  Heavy DIY (eg: weeding, lawn mowing, carpentry, digging) = 0;  Other exercises (eg: swimming, cycling, keep fit, bowling) = 0;  None of the above = 1 |

**Table S3** Disease definitions (ICD-10) used in the UK biobank study

| Health condition | ICD-10 (Field:41270) |
| --- | --- |
| Coronary artery disease | I21, I22, I23, I24.1, I25.2 |
| Ischemic stroke | I63, I64 |
| Heart failure | I50 |
| CVD | I21, I22, I23, I24.1, I25.2, I63, I64, I50 |
| Diabetes | E10-E14 |
| Hypertension | I10 |
| Hyperlipidemia | E78.0, E78.1, E78.2, E78.3, E78.4, E78.5 |
| Renal Impairment | N03, N04, N05, N08.1,  NO8.3, NO8.5, N11.1,  N11.8, N11.9, N14, N18.2,  N18.3, N18.4, N18.5,  N18.6, N18.9, N19 |

**Table S4:** Baseline characteristics of participants included or excluded from analyses due to missing information

|  | Mortality Cohort | |  | CVD cohort | |
| --- | --- | --- | --- | --- | --- |
|  | Included  participants (n=349,456) | Excluded participants (n=152,676) |  | Included  participants (n=329,197) | Excluded participants (n=172,935) |
| Age | 56.50 (8.00) | 56.60 (8.31) |  | 56.18 (7.99) | 57.21 (8.25) |
| Male | 161,670 (46.3) | 67,305 (44.1) |  | 147,956 (44.9) | 81,019 (46.8) |
| BMI, kg/m^2^ | 27.37 (4.68) | 27.58 (5.08) |  | 27.26 (4.63) | 27.78 (5.10) |
| Townsend deprivation index | -1.53 (2.95) | -0.75 (3.35) |  | -1.57 (2.92) | -0.77 (3.34) |
| Drinking frequency |  |  |  |  |  |
| < 3 times per week | 188,188 (53.9) | 96,872 (63.1) |  | 176,424 (53.6) | 108,636 (62.5) |
| ≥ 3 times per week | 161,268 (46.1) | 55,804 (36.9) |  | 152,773 (46.4) | 64,299 (37.5) |
| Smoking status |  |  |  |  |  |
| Never | 187,477 (53.6) | 85,850 (57.3) |  | 179,848 (54.6) | 93,479 (55.0) |
| Previous | 126,438 (36.2) | 46,482 (31.0) |  | 116,257 (35.3) | 56,663 (33.3) |
| Current | 35,541 (10.2) | 17,396 (11.6) |  | 33,092 (10.1) | 19,845 (11.7) |
| Sleep |  |  |  |  |  |
| Normal | 261,045 (74.7) | 104,487 (70.4) |  | 246,997 (75.0) | 118,535 (70.3) |
| Lack | 82,958 (23.7) | 40,191 (27.1) |  | 77,481 (23.5) | 45,668 (27.1) |
| Excess | 5,453 (1.6) | 3,787 (2.6) |  | 4,719 (1.4) | 4,521 (2.7) |
| Hypertension | 89,457 (25.6) | 45,089 (29.5) |  | 76,986 (23.4) | 57,560 (33.3) |
| Diabetes | 15,433 (4.4) | 10,866 (7.1) |  | 12,293 (3.7) | 14,006 (8.1) |
| Hyperlipidaemia | 50112 (14.3) | 25286 (16.6) |  | 38,556 (11.7) | 36,842 (21.3) |
| Renal impairment | 5,030 (1.4) | 2,942 (1.9) |  | 3,947 (1.2) | 4,025 (2.3) |
| Lung_Function |  |  |  |  |  |
| Normal | 256,047 (73.3) | 2,311 (64.1) |  | 244,038 (74.1) | 14,320 (60.0) |
| PRISm | 38,257 (10.9) | 577 (16.0) |  | 34651 (10.5) | 4,183 (17.5) |
| AL | 55,152 (15.8) | 718 (19.9) |  | 50,508 (15.3) | 5,362 (22.5) |
| FEV_1_, L | 2.85 (0.78) | 2.60 (0.77) |  | 2.87 (0.78) | 2.64 (0.74) |
| FVC, L | 3.78 (0.98) | 3.48 (0.95) |  | 3.79 (0.99) | 3.56 (0.93) |
| FEV_1_/FVC | 0.75 (0.07) | 0.75 (0.08) |  | 0.76 (0.07) | 0.74 (0.08) |
| FEV_1_% | 92.80 (16.61) | 88.73 (18.71) |  | 93.18 (16.45) | 87.04 (18.14) |
| FVC% | 96.75 (15.33) | 93.48 (17.09) |  | 97.10 (15.21) | 91.55 (16.44) |
| PhenoAgeAccel | -0.01 (4.52) | 1.10 (5.41) |  | -0.13 (4.41) | 1.83 (5.62) |
| PhenoAgeAccel (quartile) |  |  |  |  |  |
| Q1 | 72,438 (25.0) | 639 (21.7) |  | 69,790 (25.6) | 3,287 (16.7) |
| Q2 | 72,477 (25.0) | 599 (20.3) |  | 69,199 (25.4) | 3,877 (19.6) |
| Q3 | 72,362 (25.0) | 713 (24.2) |  | 68,157 (25.0) | 4,918 (24.9) |
| Q4 | 72,081 (24.9) | 997 (33.8) |  | 65,424 (24.0) | 7,654 (38.8) |
| Serum glucose, mmol/L | 5.10 (1.16) | 5.19 (1.43) |  | 5.08 (1.11) | 5.22 (1.47) |
| Mean cell volume, fL | 82.83 (5.22) | 82.95 (5.54) |  | 82.83 (5.20) | 82.95 (5.53) |
| White blood cell count, 10^9/L | 6.85 (2.12) | 6.97 (2.12) |  | 6.82 (2.11) | 7.02 (2.13) |
| Lymphocyte, % | 28.84 (7.29) | 29.09 (7.97) |  | 28.93 (7.27) | 28.88 (7.94) |
| Red cell distribution width, % | 13.45 (0.93) | 13.60 (1.10) |  | 13.44 (0.92) | 13.60 (1.10) |
| Creatine, μmol/L | 72.35 (17.12) | 72.20 (21.58) |  | 71.88 (16.26) | 73.16 (22.38) |
| Median CRP ( IQR), mg/dL | 0.13 (0.06, 0.26) | 0.15 (0.07, 0.31) |  | 0.13 (0.06, 0.26) | 0.15 (0.07, 0.31) |
| Alkaline phosphatase, U/L | 82.91 (25.80) | 85.50 (27.88) |  | 82.67 (25.68) | 85.65 (27.84) |
| Albumin, g/L | 45.29 (2.59) | 45.01 (2.70) |  | 45.32 (2.59) | 45.00 (2.70) |
| Frailty scores | 0.57 (0.81) | 0.81 (0.98) |  | 0.55 (0.78) | 0.84 (0.99) |
| Frailty phenotype |  |  |  |  |  |
| Normal | 194,191 (58.3) | 63,721 (47.5) |  | 186,389 (59.3) | 71,523 (46.7) |
| Pre-frail | 128,655 (38.6) | 61,130 (45.6) |  | 119,375 (38.0) | 70,410 (46.0) |
| Frail | 10,448 (3.1) | 9,196 (6.9) |  | 8,498 (2.7) | 11,146 (7.3) |
| Weight loss | 51,847 (15.1) | 23,933 (16.2) |  | 48,278 (14.9) | 27,502 (16.4) |
| Tiredness | 37,700 (11.1) | 24,430 (16.9) |  | 34,302 (10.7) | 27,828 (16.9) |
| Slow walking speed | 22,891 (6.6) | 17,989 (12.0) |  | 18,628 (5.7) | 22,252 (13.1) |
| Weakness | 62,420 (17.9) | 39,406 (26.2) |  | 56,174 (17.1) | 45,652 (26.7) |
| Physical inactivity | 26,905 (7.7) | 18,719 (12.8) |  | 24,254 (7.4) | 21,370 (12.9) |

Data are reported as mean (SD) or n (%), unless stated otherwise

Abbreviation: PRISm, preserved ratio impaired spirometry; AL, airflow limitation; CVD, cardiovascular disease.

**Table S5** Baseline characteristics of participants based on pre-existing CVD status

|  | CVD | |  |
| --- | --- | --- | --- |
|  | No (n=329197) | Yes (n=20259) | p |
| Age | 56.18 (7.99) | 61.77 (6.03) | < 0.001 |
| Male | 147956 (44.9) | 13714 (67.7) | < 0.001 |
| BMI, kg/m^2^ | 27.26 (4.63) | 29.22 (4.98) | < 0.001 |
| Townsend deprivation index | -1.57 (2.92) | -0.94 (3.25) | < 0.001 |
| Drinking frequency |  |  | < 0.001 |
| < 3 times per week | 176424 (53.6) | 11764 (58.1) |  |
| ≥ 3 times per week | 152773 (46.4) | 8495 (41.9) |  |
| Smoking status |  |  | < 0.001 |
| Never | 179848 (54.6) | 7629 (37.7) |  |
| Previous | 116257 (35.3) | 10181 (50.3) |  |
| Current | 33092 (10.1) | 2449 (12.1) |  |
| Sleep |  |  | < 0.001 |
| Normal | 246997 (75.0) | 14048 (69.3) |  |
| Lack | 77481 (23.5) | 5477 (27.0) |  |
| Excess | 4719 (1.4) | 734 (3.6) |  |
| Hypertension | 76986 (23.4) | 12471 (61.6) | < 0.001 |
| Diabetes | 12293 (3.7) | 3140 (15.5) | < 0.001 |
| Hyperlipidaemia | 38556 (11.7) | 11556 (57.0) | < 0.001 |
| Renal impairment | 3947 (1.2) | 1083 (5.3) | < 0.001 |
| Lung function phenotype |  |  | < 0.001 |
| Normal | 244038 (74.1) | 12009 (59.3) |  |
| PRISm | 34651 (10.5) | 3606 (17.8) |  |
| AL | 50508 (15.3) | 4644 (22.9) |  |
| FEV1, L | 2.87 (0.78) | 2.65 (0.74) | < 0.001 |
| FVC, L | 3.79 (0.99) | 3.58 (0.93) | < 0.001 |
| FEV_1_/FVC | 0.76 (0.07) | 0.74 (0.08) | < 0.001 |
| FEV_1_% | 93.18 (16.45) | 86.74 (18.02) | < 0.001 |
| FVC% | 97.10 (15.21) | 91.20 (16.29) | < 0.001 |
| PhenoAgeAccel | -0.13 (4.41) | 1.96 (5.65) | < 0.001 |
| PhenoAgeAccel (quartile) |  |  | < 0.001 |
| Q1 | 69790 (25.6) | 2648 (15.8) |  |
| Q2 | 69199 (25.4) | 3278 (19.5) |  |
| Q3 | 68157 (25.0) | 4205 (25.0) |  |
| Q4 | 65424 (24.0) | 6657 (39.7) |  |
| Serum glucose, mmol/L | 5.08 (1.11) | 5.46 (1.73) | < 0.001 |
| Mean cell volume, fL | 82.83 (5.20) | 82.93 (5.45) | 0.011 |
| White blood cell count, 10^9/L | 6.82 (2.11) | 7.38 (2.22) | < 0.001 |
| Lymphocyte, % | 28.93 (7.27) | 27.36 (7.51) | < 0.001 |
| Red cell distribution width, % | 13.44 (0.92) | 13.65 (1.06) | < 0.001 |
| Creatine, μmol/L | 71.88 (16.26) | 80.11 (26.49) | < 0.001 |
| Median CRP ( IQR), mg/dL | 0.24 (0.40) | 0.29 (0.49) | < 0.001 |
| Alkaline phosphatase, U/L | 82.67 (25.68) | 86.74 (27.53) | < 0.001 |
| Albumin, g/L | 45.32 (2.59) | 44.96 (2.68) | < 0.001 |
| Frailty scores | 0.55 (0.78) | 0.99 (1.08) | < 0.001 |
| Frailty phenotype |  |  | < 0.001 |
| Normal | 186389 (59.3) | 7802 (41.0) |  |
| Pre-frail | 119375 (38.0) | 9280 (48.8) |  |
| Frail | 8498 (2.7) | 1950 (10.2) |  |
| Weight loss | 48278 (14.9) | 3569 (17.9) | < 0.001 |
| Tiredness | 34302 (10.7) | 3398 (17.3) | < 0.001 |
| Slow walking speed | 18628 (5.7) | 4263 (21.3) | < 0.001 |
| Weakness | 56174 (17.1) | 6246 (30.9) | < 0.001 |
| Physical inactivity | 24254 (7.4) | 2651 (13.3) | < 0.001 |

Data are reported as mean (SD) or n (%), unless stated otherwise

Abbreviation: PRISm, preserved ratio impaired spirometry; AL, airflow limitation.

**Table S6** Association between lung function phenotypes and biological aging

|  | Model 1^a^ | | | | Model 2^b^ | | | |
| --- | --- | --- | --- | --- | --- | --- | --- | --- |
|  | PRISm | | AL | | PRISm | | AL | |
|  | *β* (95%CI) | *P* value | *β* (95%CI) | *P* value | *β* (95%CI) | *P* value | *β* (95%CI) | *P* value |
| PhnoAgeAccel | 0.826 (0.775-0.877) | ＜0.001 | 0.842 (0.798-0.887) | ＜0.001 | 0.655 (0.604-0.705) | ＜0.001 | 0.774 (0.731-0.818) | ＜0.001 |
| Frailty score | 0.166 (0.157-0.174) | ＜0.001 | 0.105 (0.098-0.113) | ＜0.001 | 0.129 (0.120-0.137) | ＜0.001 | 0.085 (0.077-0.092) | ＜0.001 |

^a^Adjusted for age, gender, BMI, and smoking status (never/former/current).

^b^Adjusted for sleep status (normal/excess/lack), deprivation index, drinking frequency (< 3 or ≥ 3 times a week), hypertension (yes/no), diabetes (yes/no), hyperlipidemia (yes/no), and renal impairment (yes/no) based on model 1.

Abbreviation: CI, confidence interval; OR: odds ratio; PRISm, preserved ratio impaired spirometry; AL, airflow limitation.

**Table S7** Associations of biological aging with mortality and CVD incidence

|  | All-cause mortality | |  | CVD | |  | IS | |  | CAD | |  | HF | |
| --- | --- | --- | --- | --- | --- | --- | --- | --- | --- | --- | --- | --- | --- | --- |
|  | HR (95%CI ) | *P* value |  | HR (95%CI ) | *P* value |  | HR (95%CI ) | *P* value |  | HR (95%CI ) | *P* value |  | HR (95%CI ) | *P* value |
| **PhenoAgeAccel** | | | | | | | | | | | | | | |
| Continuous | 1.06  (1.06-1.06) | < 0.001 |  | 1.03  (1.03-1.04) | < 0.001 |  | 1.04  (1.03-1.04) | < 0.001 |  | 1.03  (1.02-1.03) | < 0.001 |  | 1.06  (1.05-1.06) | < 0.001 |
| Quartile 1 | 1.00  (Reference) | - |  | 1.00  (Reference) | - |  | 1.00 (Reference) | - |  | 1.00 (Reference) | - |  | 1.00 (Reference) | - |
| Quartile 2 | 1.08  (1.03-1.13) | < 0.001 |  | 1.10  (1.06-1.14) | < 0.001 |  | 1.03  (0.94-1.13) | 0.497 |  | 1.10  (1.05-1.14) | < 0.001 |  | 1.21  (1.12-1.32) | < 0.001 |
| Quartile 3 | 1.23  (1.18-1.28) | < 0.001 |  | 1.15  (1.11-1.19) | < 0.001 |  | 1.21  (1.11-1.31) | < 0.001 |  | 1.13  (1.08-1.17) | < 0.001 |  | 1.33  (1.23-1.43) | < 0.001 |
| Quartile 4 | 1.87  (1.80-1.95) | < 0.001 |  | 1.40  (1.35-1.45) | < 0.001 |  | 1.47  (1.35-1.60) | < 0.001 |  | 1.32  (1.27-1.38) | < 0.001 |  | 1.95  (1.81-2.10) | < 0.001 |
| *P* trend | < 0.001 |  |  | < 0.001 |  |  | < 0.001 |  |  | < 0.001 |  |  | < 0.001 |  |
| **Frailty** | | | | | | | | | | | | | | |
| Continuous | 1.27  (1.25-1.28) | < 0.001 |  | 1.19  (1.18-1.21) | < 0.001 |  | 1.14  (1.11-1.18) | < 0.001 |  | 1.18  (1.17-1.20) | < 0.001 |  | 1.30  (1.27-1.32) | < 0.001 |
| Normal | 1.00  (Reference) | - |  | 1.00  (Reference) | - |  | 1.00 (Reference) | - |  | 1.00 (Reference) | - |  | 1.00 (Reference) | - |
| Pre-Frail | 1.29  (1.25-1.32) | < 0.001 |  | 1.22  (1.19-1.25) | < 0.001 |  | 1.17  (1.11-1.23) | < 0.001 |  | 1.21  (1.18-1.25) | < 0.001 |  | 1.35  (1.29-1.41) | < 0.001 |
| Frail | 2.20  (2.09-2.31) | < 0.001 |  | 1.77  (1.68-1.86) | < 0.001 |  | 1.48  (1.30-1.68) | < 0.001 |  | 1.71  (1.61-1.82) | < 0.001 |  | 2.28  (2.08-2.49) | < 0.001 |
| *P* trend | < 0.001 |  |  | < 0.001 |  |  | < 0.001 |  |  | < 0.001 |  |  | < 0.001 |  |

All models adjusted for age, gender, BMI, smoking status (never/former/current), sleep status (normal/excess/lack), deprivation index, drinking frequency (< 3 or ≥ 3 times a week), hypertension (yes/no), diabetes (yes/no), hyperlipidemia (yes/no), and renal impairment (yes/no).

Abbreviation: HR, hazard ratio; CI, confidence interval; CVD, cardiovascular disease; IS, ischemic stroke; CAD, coronary artery disease; HF, heart failure.

**Table S8** Association of PRISm with all-cause mortality mediated by 9 phenotypic age-related biomarkers

|  | HR (95%CI)^a^ | | |  |  |
| --- | --- | --- | --- | --- | --- |
| Mediator | Total Effect | Natural Direct Effect | Natural Indirect Effect | % Mediated | *P* value |
| Albumin | 1.38 (1.33-1.44) | 1.36 (1.31-1.42) | 1.02 (1.01-1.02) | 6.1% ( 4.5%-7.7%) | < 0.001 |
| Creatinine | 1.39 (1.33-1.44) | 1.39 (1.33-1.44) | 1.00 (1.00-1.00) | -0.6% ( -1.1%-0.0%) | 0.032 |
| Glucose | 1.39 (1.34-1.45) | 1.39 (1.33-1.45) | 1.00 (1.00-1.00) | 0.7% (0.4%-1.0%) | < 0.001 |
| Ln-CRP | 1.38 (1.33-1.44) | 1.34 (1.29-1.40) | 1.03 (1.03-1.04) | 10.8% (8.6%-13.1%) | < 0.001 |
| Lymphocyte percent | 1.38 (1.33-1.44) | 1.37 (1.32-1.42) | 1.01 (1.01-1.01) | 3.9% (2.8%-5.0%) | < 0.001 |
| Mean cell volume | 1.37 (1.32-1.43) | 1.37 (1.32-1.43) | 1.00 (1.00-1.00) | 0.3% (-0.5%-1.1%) | 0.436 |
| Red cell distribution width | 1.37 (1.32-1.43) | 1.35 (1.3-1.41) | 1.02 (1.01-1.02) | 5.8% (4.5%-7.1%) | < 0.001 |
| Alkaline phosphatase | 1.39 (1.33-1.44) | 1.37 (1.31-1.42) | 1.02 (1.01-1.02) | 6.6% (5.1%-8.1%) | < 0.001 |
| White blood cell count | 1.38 (1.33-1.44) | 1.36 (1.31-1.42) | 1.02 (1.01-1.02) | 5.4% (3.6%-7.2%) | < 0.001 |

^a^Adjusted for age, gender, BMI, smoking status (never/former/current), sleep status (normal/excess/lack), deprivation index, drinking frequency (< 3 or ≥ 3 times a week), hypertension (yes/no), diabetes (yes/no), hyperlipidemia (yes/no), and renal impairment (yes/no).

Abbreviation: HR, hazard ratio; CI, confidence interval; PRISm, preserved ratio impaired spirometry.

|  |
| --- |

**Table S9** Association of airflow limitation with all-cause mortality mediated by 9 phenotypic age-related biomarkers

|  | HR (95%CI)^a^ | | |  |  |
| --- | --- | --- | --- | --- | --- |
| Mediator | Total Effect | Natural Direct Effect | Natural Indirect Effect | % Mediated | *P* value |
| Albumin | 1.49 (1.44-1.54) | 1.47 (1.42-1.51) | 1.02 (1.02-1.02) | 5.8% (4.7%-7.0%) | <0.001 |
| Creatinine | 1.5 (1.45-1.55) | 1.50 (1.45-1.55) | 1.00 (1.00-1.00) | 0.0% (-0.1%-0.0%) | 0.49 |
| Glucose | 1.49 (1.45-1.54) | 1.50 (1.45-1.54) | 1.00 (1.00-1.00) | -0.4% (-0.6%-0.2%) | <0.001 |
| Ln-CRP | 1.47 (1.43-1.52) | 1.45 (1.40-1.49) | 1.02 (1.02-1.03) | 7.1% (6.1%-8.1%) | <0.001 |
| Lymphocyte percent | 1.48 (1.44-1.53) | 1.46 (1.42-1.51) | 1.02 (1.02-1.03) | 6.5% (5.3%-7.6%) | <0.001 |
| Mean cell volume | 1.46 (1.41-1.51) | 1.45 (1.40-1.49) | 1.01 (1.01-1.02) | 4.1% (3.2%-4.9%) | <0.001 |
| Red cell distribution width | 1.49 (1.44-1.53) | 1.46 (1.42-1.51) | 1.02 (1.02-1.02) | 5.6% (4.7%-6.4%) | <0.001 |
| Alkaline phosphatase | 1.48 (1.44-1.53) | 1.47 (1.43-1.52) | 1.01 (1.01-1.01) | 2.2% (1.6%-2.8%) | <0.001 |
| White blood cell count | 1.45 (1.41-1.50) | 1.44 (1.39-1.48) | 1.02 (1.02-1.03) | 6.8% (5.6%-8.0%) | <0.001 |

^a^Adjusted for age, gender, BMI, smoking status (never/former/current), sleep status (normal/excess/lack), deprivation index, drinking frequency (< 3 or ≥ 3 times a week), hypertension (yes/no), diabetes (yes/no), hyperlipidemia (yes/no), and renal impairment (yes/no).

Abbreviation: HR, hazard ratio; CI, confidence interval.

**Table S10** Association of PRISm with CVD incidence mediated by 9 phenotypic age-related biomarkers

|  | HR (95%CI)^a^ | | |  |  |
| --- | --- | --- | --- | --- | --- |
| Mediator | Total Effect | Natural Direct Effect | Natural Indirect Effect | % Mediated | *P* value |
| Albumin | 1.27 (1.23-1.32) | 1.26 (1.22-1.31) | 1.01 (1.01-1.02) | 5.4% (3.6%-7.1%) | < 0.001 |
| Creatinine | 1.28 (1.23-1.32) | 1.28 (1.24-1.33) | 1.00 (1.00-1.00) | -0.5% (-1.3%-0.3%) | 0.223 |
| Glucose | 1.28 (1.23-1.32) | 1.27 (1.23-1.32) | 1.00 (1.00-1.00) | 0.3% (0.0%-0.6%) | 0.047 |
| Ln-CRP | 1.28 (1.24-1.33) | 1.25 (1.21-1.30) | 1.02 (1.01-1.02) | 7.9% (5.3%-10.5%) | < 0.001 |
| Lymphocyte percent | 1.27 (1.23-1.32) | 1.27 (1.23-1.31) | 1.00 (1.00-1.01) | 2.2% (1.2%-3.1%) | < 0.001 |
| Mean cell volume | 1.27 (1.23-1.32) | 1.27 (1.23-1.32) | 1.00 (1.00-1.00) | 0.0% (-0.2%-0.2%) | 0.952 |
| Red cell distribution width | 1.27 (1.23-1.32) | 1.27 (1.22-1.31) | 1.01 (1.00-1.01) | 2.7% (1.7%-3.8%) | < 0.001 |
| Alkaline phosphatase | 1.28 (1.23-1.32) | 1.26 (1.22-1.31) | 1.01 (1.01-1.02) | 5.8% (4.0%-7.5%) | < 0.001 |
| White blood cell count | 1.27 (1.23-1.32) | 1.26 (1.22-1.31) | 1.01 (1.01-1.02) | 5.0% 2.9%-7.2%) | < 0.001 |

^a^Adjusted for age, gender, BMI, smoking status (never/former/current), sleep status (normal/excess/lack), deprivation index, drinking frequency (< 3 or ≥ 3 times a week), hypertension (yes/no), diabetes (yes/no), hyperlipidemia (yes/no), and renal impairment (yes/no).

Abbreviation: HR, hazard ratio; CI, confidence interval.

**Table S11** Association of airflow limitation with CVD incidence mediated by 9 phenotypic age-related biomarkers

|  | HR (95%CI)^a^ | | |  |  |
| --- | --- | --- | --- | --- | --- |
| Mediator | Total Effect | Natural Direct  Effect | Natural Indirect  Effect | % Mediated | *P* value |
| Albumin | 1.22 (1.18-1.25) | 1.20 (1.17-1.24) | 1.01 (1.01-1.01) | 6.2% (4.0%-8.3%) | < 0.001 |
| Creatinine | 1.23 (1.19-1.26) | 1.23 (1.19-1.27) | 1.00 (1.00-1.00) | 0.1% (-0.1%-0.2%) | 0.436 |
| Glucose | 1.22 (1.18-1.26) | 1.22 (1.18-1.26) | 1.00 (1.00-1.00) | -0.1% (-0.4%-0.2%) | 0.346 |
| Ln-CRP | 1.21 (1.17-1.24) | 1.19 (1.16-1.23) | 1.02 (1.01-1.02) | 9.3% (7.2%-11.5%) | < 0.001 |
| Lymphocyte percent | 1.21 (1.18-1.25) | 1.21 (1.17-1.24) | 1.01 (1.00-1.01) | 3.4% (1.7%-5.1%) | < 0.001 |
| Mean cell volume | 1.20 (1.17-1.24) | 1.20 (1.16-1.24) | 1.00 (1.00-1.01) | 2.4% (1.2%-3.5%) | < 0.001 |
| Red cell distribution width | 1.21 (1.18-1.25) | 1.21 (1.17-1.24) | 1.01 (1.01-1.01) | 5.2% (3.8%-6.7%) | < 0.001 |
| Alkaline phosphatase | 1.21 (1.18-1.25) | 1.21 (1.17-1.24) | 1.00 (1.00-1.01) | 2.5% (1.6%-3.5%) | < 0.001 |
| White blood cell count | 1.20 (1.17-1.24) | 1.19 (1.16-1.23) | 1.01 (1.01-1.02) | 7.6% (5.3%-9.8%) | < 0.001 |

^a^Adjusted for age, gender, BMI, smoking status (never/former/current), sleep status (normal/excess/lack), deprivation index; drinking frequency (< 3 or ≥ 3 times a week), hypertension (yes/no), diabetes (yes/no), hyperlipidemia (yes/no), and renal impairment (yes/no).

Abbreviation: HR, hazard ratio; CI, confidence interval; PRISm, preserved ratio impaired spirometry.

**Table S12** Association of PRISm with all-cause mortality and CVD incidence mediated by each frailty component

|  |  | HR (95%CI)^a^ | | |  |  |
| --- | --- | --- | --- | --- | --- | --- |
| Outcome | Mediator | Total Effect | Natural Direct Effect | Natural Indirect Effect | % Mediated | *P* value |
| All-cause mortality | Weight loss | 1.38 (1.33-1.43) | 1.39 (1.34-1.44) | 1.00 (1.00-1.00) | -0.9% (-1.6%-0.2%) | 0.011 |
|  | Exhaustion | 1.38 (1.33-1.43) | 1.37 (1.32-1.42) | 1.00 (1.00-1.01) | 1.6% (1.0%-2.2%) | < 0.001 |
|  | Slow walking speed | 1.34 (1.29-1.39) | 1.32 (1.27-1.38) | 1.01 (1.01-1.02) | 5.8% (4.4%-7.1%) | < 0.001 |
|  | Weakness | 1.38 (1.33-1.43) | 1.36 (1.31-1.41) | 1.01 (1.01-1.02) | 5.2% (3.2%-7.1%) | < 0.001 |
|  | Physical inactivity | 1.38 (1.33-1.43) | 1.37 (1.32-1.42) | 1.01 (1.01-1.01) | 2.9% (2.0%-3.8%) | < 0.001 |
| CVD | Weight loss | 1.28 (1.24-1.32) | 1.28 (1.24-1.32) | 1.00 (1.00-1.00) | -0.1% (-0.9%-0.6%) | 0.729 |
|  | Exhaustion | 1.27 (1.23-1.31) | 1.26 (1.22-1.31) | 1.00 (1.00-1.01) | 1.8% (1.1%-2.6%) | < 0.001 |
|  | Slow walking speed | 1.27 (1.23-1.32) | 1.26 (1.22-1.31) | 1.01 (1.00-1.01) | 2.6% (1.7%-3.5%) | < 0.001 |
|  | Weakness | 1.28 (1.24-1.32) | 1.26 (1.22-1.31) | 1.01 (1.00-1.01) | 3.5% (1.4%-5.7%) | 0.001 |
|  | Physical inactivity | 1.28 (1.24-1.32) | 1.28 (1.23-1.32) | 1.00 (1.00-1.00) | 1.0% (0.3%-1.7%) | 0.005 |

^a^Adjusted for age, gender, BMI, smoking status (never/former/current), sleep status (normal/excess/lack), deprivation index; drinking frequency (< 3 or ≥ 3 times a week), hypertension (yes/no), diabetes (yes/no), hyperlipidemia (yes/no), and renal impairment (yes/no).

Abbreviation: HR, hazard ratio; CI, confidence interval; PRISm, preserved ratio impaired spirometry.

**Table S13** Association of airflow limitation with all-cause mortality and CVD incidence mediated by each frailty component

|  |  | HR (95%CI)^a^ | | |  |  |
| --- | --- | --- | --- | --- | --- | --- |
| Outcome | Mediator | Total Effect | Natural Direct Effect | Natural Indirect Effect | % Mediated | *P* value |
| All-cause mortailty | Weight loss | 1.48 (1.44-1.52) | 1.48 (1.44-1.52) | 1.00 (1.00-1.00) | -0.1% (-0.3%-0.0%) | 0.180 |
|  | Exhaustion | 1.48 (1.43-1.52) | 1.47 (1.43-1.52) | 1.00 (1.00-1.01) | 1.2% (0.8%-1.6%) | < 0.001 |
|  | Slow walking speed | 1.44 (1.40-1.49) | 1.42 (1.38-1.47) | 1.02 (1.01-1.02) | 5.2% (4.3%-6.1%) | < 0.001 |
|  | Weakness | 1.48 (1.43-1.52) | 1.47 (1.43-1.51) | 1.00 (1.00-1.01) | 1.3% (0.9%-1.8%) | < 0.001 |
|  | Physical inactivity | 1.46 (1.42-1.51) | 1.46 (1.41-1.50) | 1.01 (1.01-1.01) | 2.9% (2.3%-3.6%) | < 0.001 |
| CVD | Weight loss | 1.22 (1.19-1.25) | 1.22 (1.19-1.25) | 1.00 (1.00-1.00) | -0.1% (-0.3%-0.1%) | 0.459 |
|  | Exhaustion | 1.22 (1.18-1.25) | 1.21 (1.18-1.25) | 1.00 (1.00-1.00) | 2.0% (1.2%-2.7%) | < 0.001 |
|  | Slow walking speed | 1.2 (1.17-1.24) | 1.19 (1.16-1.23) | 1.01 (1.01-1.01) | 5.3% (3.9%-6.6%) | < 0.001 |
|  | Weakness | 1.22 (1.18-1.25) | 1.21 (1.18-1.25) | 1.00 (1.00-1.00) | 1.6% (0.9%-2.3%) | < 0.001 |
|  | Physical inactivity | 1.21 (1.18-1.25) | 1.21 (1.17-1.24) | 1.01 (1.00-1.01) | 3.3% (2.3%-4.4%) | < 0.001 |

^a^Adjusted for age, gender, BMI, smoking status (never/former/current), sleep status (normal/excess/lack), deprivation index; drinking frequency (< 3 or ≥ 3 times a week), hypertension (yes/no), diabetes (yes/no), hyperlipidemia (yes/no), and renal impairment (yes/no).

Abbreviation: HR, hazard ratio; CI, confidence interval.

**Table S14** Associations of PhenoAgeAccel with mortality and CVD incidence by lung function phenotypes

|  | All-cause mortality | |  | CVD | |
| --- | --- | --- | --- | --- | --- |
|  | HR (95%CI) | *P* value |  | HR (95%CI) | *P* value |
| **Normal** |  |  |  |  |  |
| Q1 | 1.00 (Reference) | - |  | 1.00 (Reference) | - |
| Q2 | 1.06 (1.00-1.12) | 0.038 |  | 1.09 (1.04-1.13) | < 0.001 |
| Q3 | 1.20 (1.14-1.26) | < 0.001 |  | 1.13 (1.08-1.18) | < 0.001 |
| Q4 | 1.68 (1.59-1.76) | < 0.001 |  | 1.34 (1.28-1.40) | < 0.001 |
| **PRISm** |  |  |  |  |  |
| Q1 | 1.00 (Reference) | - |  | 1.00 (Reference) | - |
| Q2 | 0.97 (0.85-1.11) | 0.682 |  | 1.16 (1.04-1.30) | 0.007 |
| Q3 | 1.14 (1.01-1.29) | 0.038 |  | 1.24 (1.11-1.38) | < 0.001 |
| Q4 | 1.85 (1.65-2.07) | < 0.001 |  | 1.56 (1.41-1.73) | < 0.001 |
| **AL** |  |  |  |  |  |
| Q1 | 1.00 (Reference) | - |  | 1.00 (Reference) | - |
| Q2 | 1.17 (1.06-1.29) | 0.001 |  | 1.02 (0.93-1.11) | 0.727 |
| Q3 | 1.29 (1.18-1.41) | < 0.001 |  | 1.09 (1.01-1.18) | 0.038 |
| Q4 | 2.08 (1.91-2.26) | < 0.001 |  | 1.37 (1.26-1.48) | < 0.001 |

All models adjusted for age, gender, BMI, smoking status (never/former/current), sleep status (normal/excess/lack), deprivation index, drinking frequency (< 3 or ≥ 3 times a week), hypertension (yes/no), diabetes (yes/no), hyperlipidemia (yes/no), and renal impairment (yes/no).

Abbreviation: CI, confidence interval; CVD, cardiovascular disease; HR, hazard ratio; PRISm, preserved ratio impaired spirometry; AL, airflow limitation.

**Table S15** Associations of frailty with mortality and CVD incidence by lung function phenotypes

|  | All-cause mortality | |  | CVD | |
| --- | --- | --- | --- | --- | --- |
|  | HR (95%CI) | *P* value |  | HR (95%CI) | *P* value |
| **Normal** |  |  |  |  |  |
| Normal | 1.00 (Reference) | - |  | 1.00 (Reference) | - |
| Pre-frail | 1.21 (1.17-1.25) | < 0.001 |  | 1.20 (1.17-1.24) | < 0.001 |
| Frail | 1.90 (1.76-2.05) | < 0.001 |  | 1.71 (1.59-1.84) | < 0.001 |
| **PRISm** |  |  |  |  |  |
| Normal | 1.00 (Reference) | - |  | 1.00 (Reference) | - |
| Pre-frail | 1.36 (1.26-1.46) | < 0.001 |  | 1.16 (1.08-1.23) | < 0.001 |
| Frail | 2.19 (1.96-2.45) | < 0.001 |  | 1.59 (1.42-1.78) | < 0.001 |
| **AL** |  |  |  |  |  |
| Normal | 1.00 (Reference) | - |  | 1.00 (Reference) | - |
| Pre-frail | 1.35 (1.29-1.42) | < 0.001 |  | 1.26 (1.20-1.33) | < 0.001 |
| Frail | 2.35 (2.15-2.56) | < 0.001 |  | 1.87 (1.68-2.07) | < 0.001 |

All models adjusted for age, gender, BMI, smoking status (never/former/current), sleep status (normal/excess/lack), deprivation index, drinking frequency (< 3 or ≥ 3 times a week), hypertension (yes/no), diabetes (yes/no), hyperlipidemia (yes/no), and renal impairment (yes/no).

Abbreviation: CI, confidence interval; CVD, cardiovascular disease; HR, hazard ratio; PRISm, preserved ratio impaired spirometry; AL, airflow limitation.

**Table S16** The joint association of lung function phenotypes and PhnoAgeAccel quartiles with the risk of mortality and CVD incidence

|  | All-cause mortality | |  | CVD | |
| --- | --- | --- | --- | --- | --- |
|  | HR (95%CI) | *P* value |  | HR (95%CI) | *P* value |
| Normal |  |  |  |  |  |
| Q1 | 1.00 (Reference) | - |  | 1.00 (Reference) | - |
| Q2 | 1.06 (1.01-1.12) | 0.023 |  | 1.10 (1.05-1.15) | < 0.001 |
| Q3 | 1.21 (1.14-1.27) | < 0.001 |  | 1.15 (1.10-1.20) | < 0.001 |
| Q4 | 1.68 (1.60-1.76) | < 0.001 |  | 1.36 (1.30-1.42) | < 0.001 |
| PRISm |  |  |  |  |  |
| Q1 | 1.32 (1.19-1.46) | < 0.001 |  | 1.20 (1.10-1.32) | < 0.001 |
| Q2 | 1.29 (1.17-1.42) | < 0.001 |  | 1.37 (1.26-1.48) | < 0.001 |
| Q3 | 1.51 (1.39-1.64) | < 0.001 |  | 1.43 (1.33-1.53) | < 0.001 |
| Q4 | 2.46 (2.30-2.62) | < 0.001 |  | 1.76 (1.66-1.87) | < 0.001 |
| AL |  |  |  |  |  |
| Q1 | 1.26 (1.16-1.36) | < 0.001 |  | 1.24 (1.15-1.33) | < 0.001 |
| Q2 | 1.46 (1.36-1.57) | < 0.001 |  | 1.24 (1.16-1.32) | < 0.001 |
| Q3 | 1.62 (1.51-1.73) | < 0.001 |  | 1.33 (1.25-1.41) | < 0.001 |
| Q4 | 2.65 (2.51-2.79) | < 0.001 |  | 1.68 (1.59-1.77) | < 0.001 |

All models adjusted for age, gender, BMI, smoking status (never/former/current), sleep status (normal/excess/lack), deprivation index, drinking frequency (< 3 or ≥ 3 times a week), hypertension (yes/no), diabetes (yes/no), hyperlipidemia (yes/no), and renal impairment (yes/no).

Abbreviation: CI, confidence interval; CVD, cardiovascular disease; HR, hazard ratio; PRISm, preserved ratio impaired spirometry; AL, airflow limitation.

**Table S17** The joint association of lung function phenotypes and frailty status with the risk of mortality and CVD incidence

|  | All-cause mortality | |  | CVD | |
| --- | --- | --- | --- | --- | --- |
|  | HR (95%CI) | *P* value |  | HR (95%CI) | *P* value |
| **Normal** |  |  |  |  |  |
| Normal | 1.00 (Reference) | - |  | 1.00 (Reference) | - |
| Pre-frail | 1.22 (1.18-1.26) | < 0.001 |  | 1.20 (1.17-1.24) | < 0.001 |
| Frail | 1.92 (1.78-2.07) | < 0.001 |  | 1.71 (1.59-1.84) | < 0.001 |
| **PRISm** |  |  |  |  |  |
| Normal | 1.26 (1.18-1.33) | < 0.001 |  | 1.28 (1.22-1.35) | < 0.001 |
| Pre-frail | 1.71 (1.62-1.79) | < 0.001 |  | 1.48 (1.42-1.55) | < 0.001 |
| Frail | 2.78 (2.54-3.04) | < 0.001 |  | 2.03 (1.84-2.24) | < 0.001 |
| **AL** |  |  |  |  |  |
| Normal | 1.36 (1.31-1.42) | < 0.001 |  | 1.17 (1.12-1.21) | < 0.001 |
| Pre-frail | 1.84 (1.77-1.92) | < 0.001 |  | 1.49 (1.43-1.55) | < 0.001 |
| Frail | 3.23 (2.99-3.50) | < 0.001 |  | 2.24 (2.04-2.46) | < 0.001 |

All models adjusted for age, gender, BMI, smoking status (never/former/current), sleep status (normal/excess/lack), deprivation index, drinking frequency (< 3 or ≥ 3 times a week), hypertension (yes/no), diabetes (yes/no), hyperlipidemia (yes/no), and renal impairment (yes/no).

Abbreviation: CI, confidence interval; CVD, cardiovascular disease; HR, hazard ratio; PRISm, preserved ratio impaired spirometry; AL, airflow limitation.

**Table S18** Hazard ratios (95%CI) of lung function phenotypes for all-cause mortality and CVD incidence by multiple imputation

|  | Normal | PRISm | AL |
| --- | --- | --- | --- |
| **All-cause mortality** | | | |
| Model 1^a^ | 1.00 (Reference) | 1.50 (1.45-1.55) | 1.55 (1.51-1.59) |
| Model 2^b^ | 1.00 (Reference) | 1.40 (1.35-1.45) | 1.50 (1.46-1.54) |
| **CVD** | | | |
| Model 1 | 1.00 (Reference) | 1.34 (1.30-1.38) | 1.25 (1.22-1.28) |
| Model 2 | 1.00 (Reference) | 1.27 (1.23-1.31) | 1.22 (1.19-1.26) |

^a^Adjusted for age, gender, BMI, and smoking status (never/former/current).

^b^Adjusted for sleep status (normal/excess/lack), deprivation index, drinking frequency (< 3 or ≥ 3 times a week), hypertension (yes/no), diabetes (yes/no), hyperlipidemia (yes/no), and renal impairment (yes/no) based on model 1.

Abbreviation: CI, confidence interval; PRISm, preserved ratio impaired spirometry; AL, airflow limitation; CVD, cardiovascular disease.

**Table S19** The mediating effect of biological aging on the association between lung function phenotypes and primary outcomes by multiple imputation

|  | Total Effect | Natural Direct Effect | Natural Indirect Effect | % Mediated | *P* value |
| --- | --- | --- | --- | --- | --- |
| **All-cause death** | | | | | |
| **PhenoAgeAccel** | | | | | |
| PRISm | 1.38 (1.33-1.44) | 1.33 (1.28-1.39) | 1.04 (1.03-1.04) | 12.9% (10.9%-14.9%) | < 0.001 |
| AL | 1.47 (1.43-1.52) | 1.41 (1.37-1.46) | 1.05 (1.05-1.06) | 15.7% (14.0%-17.3%) | < 0.001 |
| **Frailty** | | | | | |
| PRISm | 1.36 (1.31-1.41) | 1.32 (1.27-1.37) | 1.03 (1.03-1.04) | 12.1% (9.9%-14.3%) | < 0.001 |
| AL | 1.46 (1.42-1.51) | 1.44 (1.40-1.48) | 1.02 (1.02-1.03) | 7.0% (6.0%-8.0%) | < 0.001 |
| **CVD** | | | | | |
| **PhenoAgeAccel** | | | | | |
| PRISm | 1.27 (1.23-1.32) | 1.25 (1.21-1.30) | 1.02 (1.02-1.02) | 8.8% (6.7%-10.9%) | < 0.001 |
| AL | 1.21 (1.17-1.24) | 1.18 (1.15-1.22) | 1.03 (1.02-1.03) | 15.7% (12.4%-19.0%) | < 0.001 |
| **Frailty** | | | | | |
| PRISm | 1.28 (1.24-1.33) | 1.26 (1.21-1.30) | 1.02 (1.01-1.02) | 7.8% (5.8%-9.8%) | < 0.001 |
| AL | 1.22 (1.18-1.25) | 1.20 (1.17-1.23) | 1.02 (1.01-1.02) | 8.6% (6.8%-10.4%) | < 0.001 |

All models adjusted for age, gender, BMI, smoking status (never/former/current), sleep status (normal/excess/lack), deprivation index, drinking frequency (< 3 or ≥ 3 times a week), hypertension (yes/no), diabetes (yes/no), hyperlipidemia (yes/no), and renal impairment (yes/no).

Abbreviation: HR, hazard ratio; CI, confidence interval; CVD, cardiovascular disease; PRISm, preserved ratio impaired spirometry; AL, airflow limitation.

**Table S20** Hazard ratios (95%CI) of lung function phenotypes for all-cause mortality and CVD incidence by random forest imputation

|  | Normal | PRISm | AL |
| --- | --- | --- | --- |
| **All-cause mortality** | | | |
| Model 1^a^ | 1.00 (Reference) | 1.50 (1.45-1.55) | 1.55 (1.51-1.59) |
| Model 2^b^ | 1.00 (Reference) | 1.40 (1.35-1.45) | 1.50 (1.46-1.54) |
| **CVD** | | | |
| Model 1 | 1.00 (Reference) | 1.34 (1.30-1.38) | 1.25 (1.22-1.28) |
| Model 2 | 1.00 (Reference) | 1.27 (1.23-1.31) | 1.22 (1.19-1.26) |

^a^Adjusted for age, gender, BMI, and smoking status (never/former/current).

^b^Adjusted for sleep status (normal/excess/lack), deprivation index, drinking frequency (< 3 or ≥ 3 times a week), hypertension (yes/no), diabetes (yes/no), hyperlipidemia (yes/no), and renal impairment (yes/no) based on model 1.

Abbreviation: CI, confidence interval; PRISm, preserved ratio impaired spirometry; AL, airflow limitation; CVD, cardiovascular disease

**Table S21** The mediating effect of biological aging on the association between lung function phenotypes and primary outcomes by random forest imputation

|  | Total Effect | Natural Direct Effect | Natural Indirect Effect | % Mediated | *P* value |
| --- | --- | --- | --- | --- | --- |
| **All-cause death** | | | | | |
| **PhenoAgeAccel** | | | | | |
| PRISm | 1.38 (1.33-1.44) | 1.33 (1.28-1.38) | 1.04 (1.03-1.04) | 12.9% (10.9%-14.9%) | < 0.001 |
| AL | 1.47 (1.43-1.52) | 1.41 (1.37-1.46) | 1.05 (1.05-1.06) | 15.6% (13.9%-17.2%) | < 0.001 |
| **Frailty** | | | | | |
| PRISm | 1.36 (1.31-1.41) | 1.32 (1.27-1.37) | 1.03 (1.03-1.04) | 12.1% (9.9%-14.3%) | < 0.001 |
| AL | 1.46 (1.42-1.51) | 1.44 (1.40-1.48) | 1.02 (1.02-1.03) | 7.0% (6.0%-8.0%) | < 0.001 |
| **CVD** | | | | | |
| **PhenoAgeAccel** | | | | | |
| PRISm | 1.27 (1.23-1.32) | 1.25 (1.21-1.3) | 1.02 (1.02-1.02) | 8.7% (6.6%-10.8%) | < 0.001 |
| AL | 1.21 (1.17-1.24) | 1.18 (1.15-1.22) | 1.03 (1.02-1.03) | 15.6% (12.3%-18.9%) | < 0.001 |
| **Frailty** | | | | | |
| PRISm | 1.28 (1.24-1.33) | 1.26 (1.21-1.3) | 1.02 (1.01-1.02) | 7.8% (5.8%-9.8%) | < 0.001 |
| AL | 1.22 (1.18-1.25) | 1.2 (1.17-1.23) | 1.02 (1.01-1.02) | 8.6% (6.8%-10.4%) | < 0.001 |

All models adjusted for age, gender, BMI, smoking status (never/former/current), sleep status (normal/excess/lack), deprivation index, drinking frequency (< 3 or ≥ 3 times a week), hypertension (yes/no), diabetes (yes/no), hyperlipidemia (yes/no), and renal impairment (yes/no).

Abbreviation: HR, hazard ratio; CI, confidence interval; CVD, cardiovascular disease; PRISm, preserved ratio impaired spirometry; AL, airflow limitation.

**Table S22** Hazard ratios (95%CI) of lung function phenotypes for all-cause mortality and CVD incidence, excluding events in the first 2 years

|  | Normal | PRISm | AL |
| --- | --- | --- | --- |
| **All-cause mortality** | | | |
| Model 1^a^ | 1.00 (Reference) | 1.47 (1.42-1.52) | 1.52 (1.48-1.57) |
| Model 2^b^ | 1.00 (Reference) | 1.37 (1.32-1.42) | 1.47 (1.43-1.51) |
| **CVD** | | | |
| Model 1 | 1.00 (Reference) | 1.32 (1.28-1.37) | 1.25 (1.22-1.29) |
| Model 2 | 1.00 (Reference) | 1.26 (1.22-1.30) | 1.23 (1.19-1.26) |

^a^Adjusted for age, gender, BMI, and smoking status (never/former/current).

^b^Adjusted for sleep status (normal/excess/lack), deprivation index, drinking frequency (< 3 or ≥ 3 times a week), hypertension (yes/no), diabetes (yes/no), hyperlipidemia (yes/no), and renal impairment (yes/no) based on model 1.

Abbreviation: CI, confidence interval; PRISm, preserved ratio impaired spirometry; AL, airflow limitation; CVD, cardiovascular disease.

**Table S23** The mediating effect of biological aging on the association between lung function phenotypes and primary outcomes , excluding events in the first 2 years

|  | Total Effect | Natural Direct Effect | Natural Indirect Effect | % Mediated | *P* value |
| --- | --- | --- | --- | --- | --- |
| **All-cause death** | | | | | |
| **PhenoAgeAccel** | | | | | |
| PRISm | 1.38 (1.33-1.44) | 1.33 (1.28-1.39) | 1.04 (1.03-1.04) | 12.9% (10.9%-14.9%) | < 0.001 |
| AL | 1.47 (1.43-1.52) | 1.41 (1.37-1.46) | 1.05 (1.05-1.06) | 15.7% (14.0%-17.3%) | < 0.001 |
| **Frailty** | | | | | |
| PRISm | 1.36 (1.31-1.41) | 1.32 (1.27-1.37) | 1.03 (1.03-1.04) | 12.1% (9.9%-14.3%) | < 0.001 |
| AL | 1.46 (1.42-1.51) | 1.44 (1.4-1.48) | 1.02 (1.02-1.03) | 7.0% (6.0%-8.0%) | < 0.001 |
| **CVD** | | | | | |
| **PhenoAgeAccel** | | | | | |
| PRISm | 1.27 (1.23-1.32) | 1.25 (1.21-1.30) | 1.02 (1.02-1.02) | 8.8% (6.7%-10.9%) | < 0.001 |
| AL | 1.21 (1.17-1.24) | 1.18 (1.15-1.22) | 1.03 (1.02-1.03) | 15.7% (12.4%-19.0%) | < 0.001 |
| **Frailty** | | | | | |
| PRISm | 1.28 (1.24-1.33) | 1.26 (1.21-1.3) | 1.02 (1.01-1.02) | 7.8% (5.8%-9.8%) | < 0.001 |
| AL | 1.22 (1.18-1.25) | 1.20 (1.17-1.23) | 1.02 (1.01-1.02) | 8.6% (6.8%-10.4%) | < 0.001 |

All models adjusted for age, gender, BMI, smoking status (never/former/current), sleep status (normal/excess/lack), deprivation index, drinking frequency (< 3 or ≥ 3 times a week), hypertension (yes/no), diabetes (yes/no), hyperlipidemia (yes/no), and renal impairment (yes/no).

Abbreviation: HR, hazard ratio; CI, confidence interval; CVD, cardiovascular disease; PRISm, preserved ratio impaired spirometry; AL, airflow limitation.

**Table S24** Hazard ratios (95%CI) of lung function phenotypes for all-cause mortality and CVD incidence before COVID-2019

|  | Normal | PRISm | AL |
| --- | --- | --- | --- |
| **All-cause mortality** | | | |
| Model 1^a^ | 1.00 (Reference) | 1.51 (1.45-1.58) | 1.58 (1.53-1.64) |
| Model 2^b^ | 1.00 (Reference) | 1.41 (1.35-1.47) | 1.52 (1.44-1.58) |
| **CVD** | | | |
| Model 1 | 1.00 (Reference) | 1.33 (1.28-1.38) | 1.24 (1.20-1.28) |
| Model 2 | 1.00 (Reference) | 1.26 (1.21-1.31) | 1.21 (1.17-1.25) |

^a^Adjusted for age, gender, BMI, and smoking status (never/former/current).

^b^Adjusted for sleep status (normal/excess/lack), deprivation index, drinking frequency (< 3 or ≥ 3 times a week), hypertension (yes/no), diabetes (yes/no), hyperlipidemia (yes/no), and renal impairment (yes/no) based on model 1.

Abbreviation: CI, confidence interval; PRISm, preserved ratio impaired spirometry; AL, airflow limitation; CVD, cardiovascular disease.

**Table S25** The mediating effect of biological aging on the association between lung function phenotypes and primary outcomes before COVID-2019

|  | Total Effect | Natural Direct Effect | Natural Indirect Effect | % Mediated | *P* value |
| --- | --- | --- | --- | --- | --- |
| **All-cause death** | | | | | |
| **PhenoAgeAccel** | | | | | |
| PRISm | 1.37 (1.30-1.44) | 1.31 (1.25-1.38) | 1.04 (1.04-1.05) | 14.6% (11.9%-17.3%) | < 0.001 |
| AL | 1.49 (1.43-1.55) | 1.42 (1.37-1.48) | 1.06 (1.05-1.06) | 16.5% (14.6%-18.5%) | < 0.001 |
| **Frailty** | | | | | |
| PRISm | 1.36 (1.30-1.43) | 1.32 (1.26-1.39) | 1.04 (1.03-1.04) | 13.2% (10.3%-16.0%) | < 0.001 |
| AL | 1.49 (1.43-1.55) | 1.46 (1.41-1.52) | 1.02 (1.02-1.03) | 7.4% (6.2%-8.5%) | < 0.001 |
| **CVD** | | | | | |
| **PhenoAgeAccel** | | | | | |
| PRISm | 1.27 (1.22-1.32) | 1.24 (1.19-1.30) | 1.02 (1.01-1.02) | 8.6% (6.2%-11.1%) | < 0.001 |
| AL | 1.19 (1.15-1.23) | 1.16 (1.12-1.20) | 1.03 (1.03-1.03) | 18.4% (13.8%-23.0%) | < 0.001 |
| **Frailty** | | | | | |
| PRISm | 1.27 (1.22-1.33) | 1.25 (1.20-1.30) | 1.02 (1.01-1.02) | 7.8% (5.5%-10.2%) | < 0.001 |
| AL | 1.19 (1.15-1.23) | 1.18 (1.14-1.22) | 1.02 (1.01-1.02) | 9.9% (7.5%-12.3%) | < 0.001 |

All models adjusted for age, gender, BMI, smoking status (never/former/current), sleep status (normal/excess/lack), deprivation index; drinking frequency (< 3 or ≥ 3 times a week), hypertension (yes/no), diabetes (yes/no), hyperlipidemia (yes/no), and renal impairment (yes/no).

Abbreviation: HR, hazard ratio; CI, confidence interval; CVD, cardiovascular disease; PRISm, preserved ratio impaired spirometry; AL, airflow limitation.

**Table S26** Hazard ratios (95%CI) of lung function phenotypes for CVD incidence excluding patients with any cardiovascular disease at baseline

|  | Normal | PRISm | AL |
| --- | --- | --- | --- |
| Model 1^a^ | 1.00 (Reference) | 1.17 (1.14-1.20) | 1.16 (1.14-1.18) |
| Model 2^b^ | 1.00 (Reference) | 1.15 (1.12-1.18) | 1.15 (1.13-1.17) |

^a^Adjusted for age, gender, BMI, and smoking status (never/former/current).

^b^Adjusted for sleep status (normal/excess/lack), deprivation index, drinking frequency (< 3 or ≥ 3 times a week), hypertension (yes/no), diabetes (yes/no), hyperlipidemia (yes/no), and renal impairment (yes/no) based on model 1.

Abbreviation: CI, confidence interval; PRISm, preserved ratio impaired spirometry; AL, airflow limitation; CVD, cardiovascular disease.

**Table S27** The mediating effect of biological aging on the association between lung function phenotypes and CVD incidence,excluding patients with any cardiovascular disease at baseline

|  | Total Effect | Natural Direct Effect | Natural Indirect Effect | % Mediated | *P* value |
| --- | --- | --- | --- | --- | --- |
| **CVD** | | | | | |
| **PhenoAgeAccel** | | | | | |
| PRISm | 1.16 (1.12-1.19) | 1.15 (1.11-1.18) | 1.01 (1.01-1.01) | 5.9% (3.5%-8.3%) | < 0.001 |
| AL | 1.15 (1.13-1.18) | 1.14 (1.11-1.16) | 1.01 (1.01-1.02) | 10.7% (7.8%-13.7%) | < 0.001 |
| **Frailty** | | | | | |
| PRISm | 1.16 (1.13-1.19) | 1.14 (1.11-1.17) | 1.01 (1.01-1.01) | 6.8% (4.5%-9.2%) | < 0.001 |
| AL | 1.14 (1.12-1.17) | 1.14 (1.11-1.16) | 1.01 (1.01-1.01) | 6.6% (4.9%-8.2%) | < 0.001 |

All models adjusted for age, gender, BMI, smoking status (never/former/current), sleep status (normal/excess/lack), deprivation index, drinking frequency (< 3 or ≥ 3 times a week), hypertension (yes/no), diabetes (yes/no), hyperlipidemia (yes/no), and renal impairment (yes/no).

Abbreviation: HR, hazard ratio; CI, confidence interval; CVD, cardiovascular disease; PRISm, preserved ratio impaired spirometry; AL, airflow limitation.

**Table S28** Hazard ratios (95%CI) of lung function phenotypes for all-cause mortality and CVD incidence, incorporating the use of specific medications

|  | Normal | PRISm | AL |
| --- | --- | --- | --- |
| **All-cause mortality** | | | |
| Model 1^a^ | 1.00 (Reference) | 1.49 (1.44-1.54) | 1.54 (1.50-1.59) |
| Model 2^b^ | 1.00 (Reference) | 1.37 (1.32-1.42) | 1.48 (1.44-1.52) |
| **CVD** | | | |
| Model 1 | 1.00 (Reference) | 1.33 (1.29-1.37) | 1.25 (1.21-1.28) |
| Model 2 | 1.00 (Reference) | 1.25 (1.22-1.30) | 1.22 (1.18-1.25) |

^a^Adjusted for age, gender, BMI, and smoking status (never/former/current).

^b^Adjusted for sleep status (normal/excess/lack), deprivation index, drinking frequency (< 3 or ≥ 3 times a week), hypertension (yes/no), diabetes (yes/no), hyperlipidemia (yes/no), renal impairment (yes/no), and the use of statins (yes/no), β-blockers (yes/no), or asprin (yes/no), based on model 1.

Abbreviation: CI, confidence interval; PRISm, preserved ratio impaired spirometry; AL, airflow limitation; CVD, cardiovascular disease.

**Table S29** The mediating effect of biological aging on the association between lung function phenotypes and primary outcomes, incorporating the use of specific medications

|  | Total Effect | Natural Direct Effect | Natural Indirect Effect | % Mediated | *P* value |
| --- | --- | --- | --- | --- | --- |
| **All-cause death** | | | | | |
| **PhenoAgeAccel** | | | | | |
| PRISm | 1.36 (1.3-1.41) | 1.31 (1.26-1.36) | 1.03 (1.03-1.04) | 12.5% (10.5%-14.6%) | < 0.001 |
| AL | 1.45 (1.40-1.50) | 1.39 (1.35-1.44) | 1.05 (1.05-1.06) | 15.9% (14.2%-17.7%) | < 0.001 |
| **Frailty** | | | | | |
| PRISm | 1.34 (1.28-1.39) | 1.3 (1.25-1.35) | 1.03 (1.03-1.03) | 11.6% (9.3%-13.9%) | < 0.001 |
| AL | 1.45 (1.40-1.49) | 1.42 (1.38-1.47) | 1.02 (1.02-1.02) | 6.6% (5.7%-7.6%) | < 0.001 |
| **CVD** | | | | | |
| **PhenoAgeAccel** | | | | | |
| PRISm | 1.26 (1.21-1.31) | 1.24 (1.19-1.28) | 1.02 (1.01-1.02) | 8.2% (6.0%-10.3%) | < 0.001 |
| AL | 1.20 (1.17-1.24) | 1.18 (1.14-1.21) | 1.03 (1.02-1.03) | 15.9% (12.4%-19.3%) | < 0.001 |
| **Frailty** | | | | | |
| PRISm | 1.27 (1.23-1.31) | 1.24 (1.20-1.29) | 1.02 (1.01-1.02) | 7.0% (5.0%-9.0%) | < 0.001 |
| AL | 1.21 (1.17-1.24) | 1.19 (1.16-1.23) | 1.01 (1.01-1.02) | 8.5% (6.6%-10.3%) | < 0.001 |

All models adjusted for age, gender, BMI, smoking status (never/former/current), sleep status (normal/excess/lack), deprivation index, drinking frequency (< 3 or ≥ 3 times a week), hypertension (yes/no), diabetes (yes/no), hyperlipidemia (yes/no), renal impairment (yes/no), and the use of statins (yes/no), β-blockers (yes/no), or asprin (yes/no).

Abbreviation: HR, hazard ratio; CI, confidence interval; CVD, cardiovascular disease; PRISm, preserved ratio impaired spirometry; AL, airflow limitation.

**
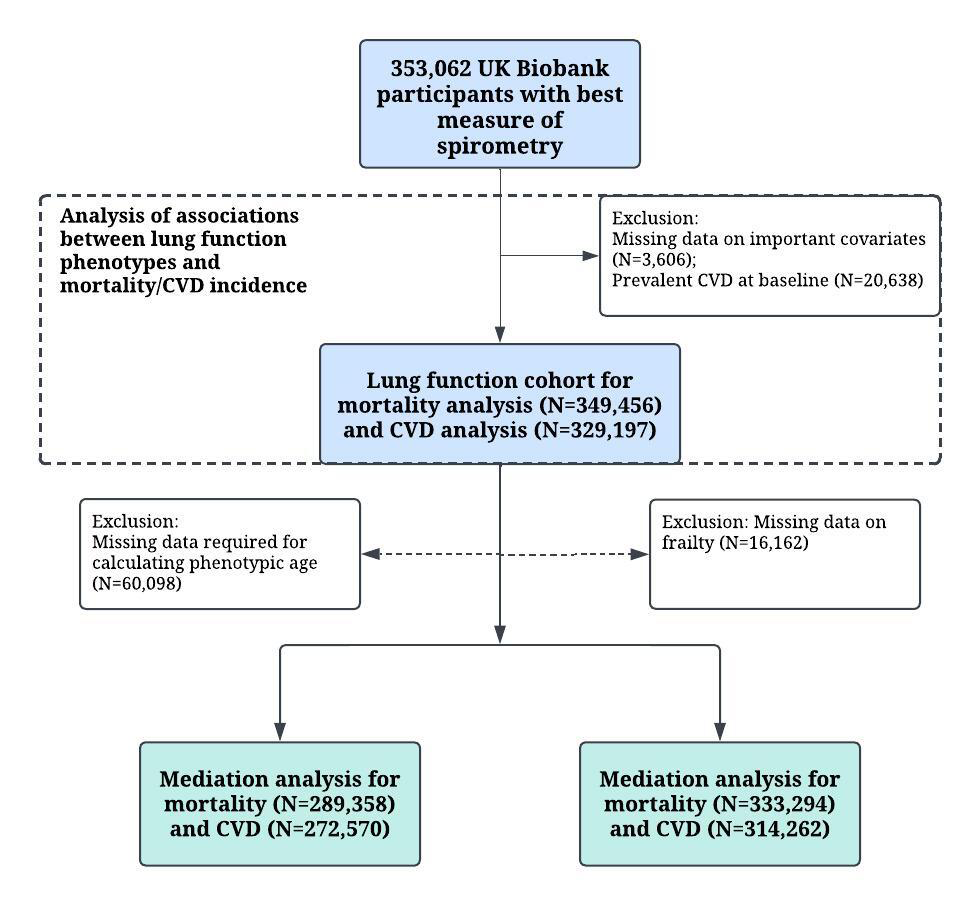
**

**Fig. S1** The flow chart of the selection of the study population

Abbreviation: CVD, cardiovascular disease.

**
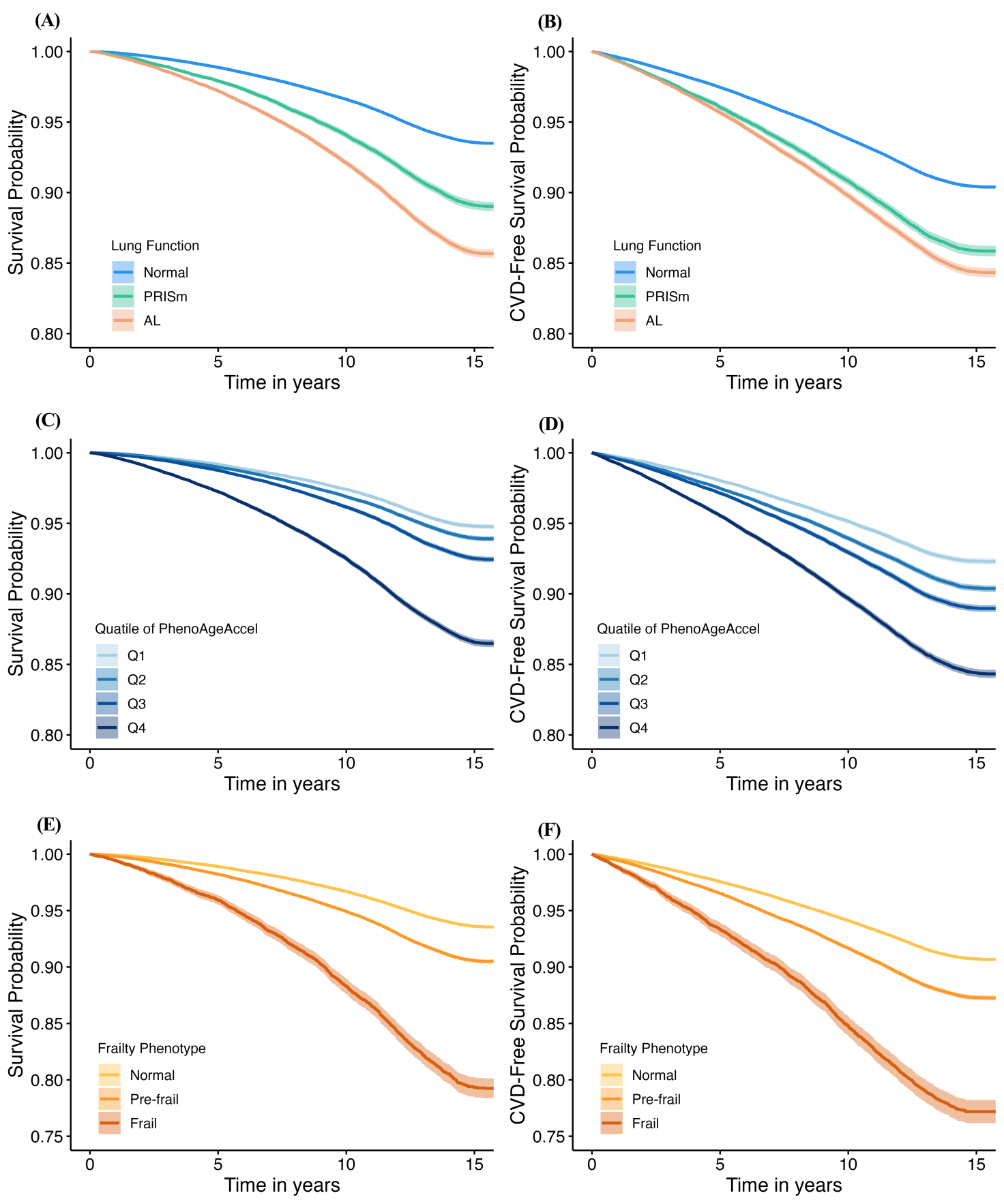
**

**Fig. S2** Kaplan-Meier survival and CVD-free survival curves stratified by lung function phenotypes, PhenoAgeAccel, and frailty phenotype

Abbreviation: PRISm, preserved ratio impaired spirometry; AL, airflow limitation; CVD, cardiovascular disease.

**
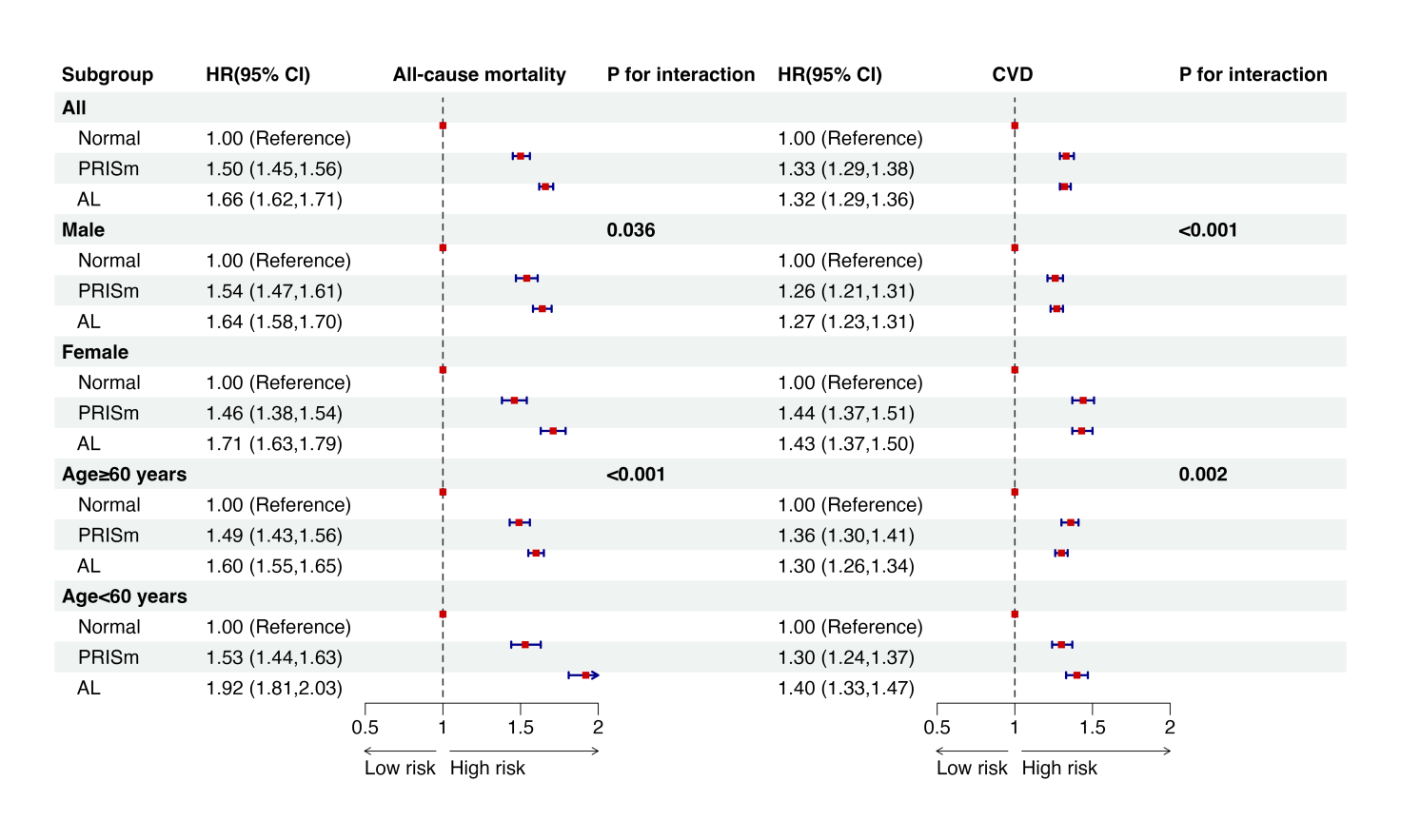
Fig. S3** Hazard ratios (HRs) for mortality/CVD incidence and lung function phenotypes

*P* for interaction indicates the test for interaction terms by subgroup variables (gender and age).

Abbreviation: CI, confidence interval; PRISm, preserved ratio impaired spirometry; AL, airflow limitation.

**
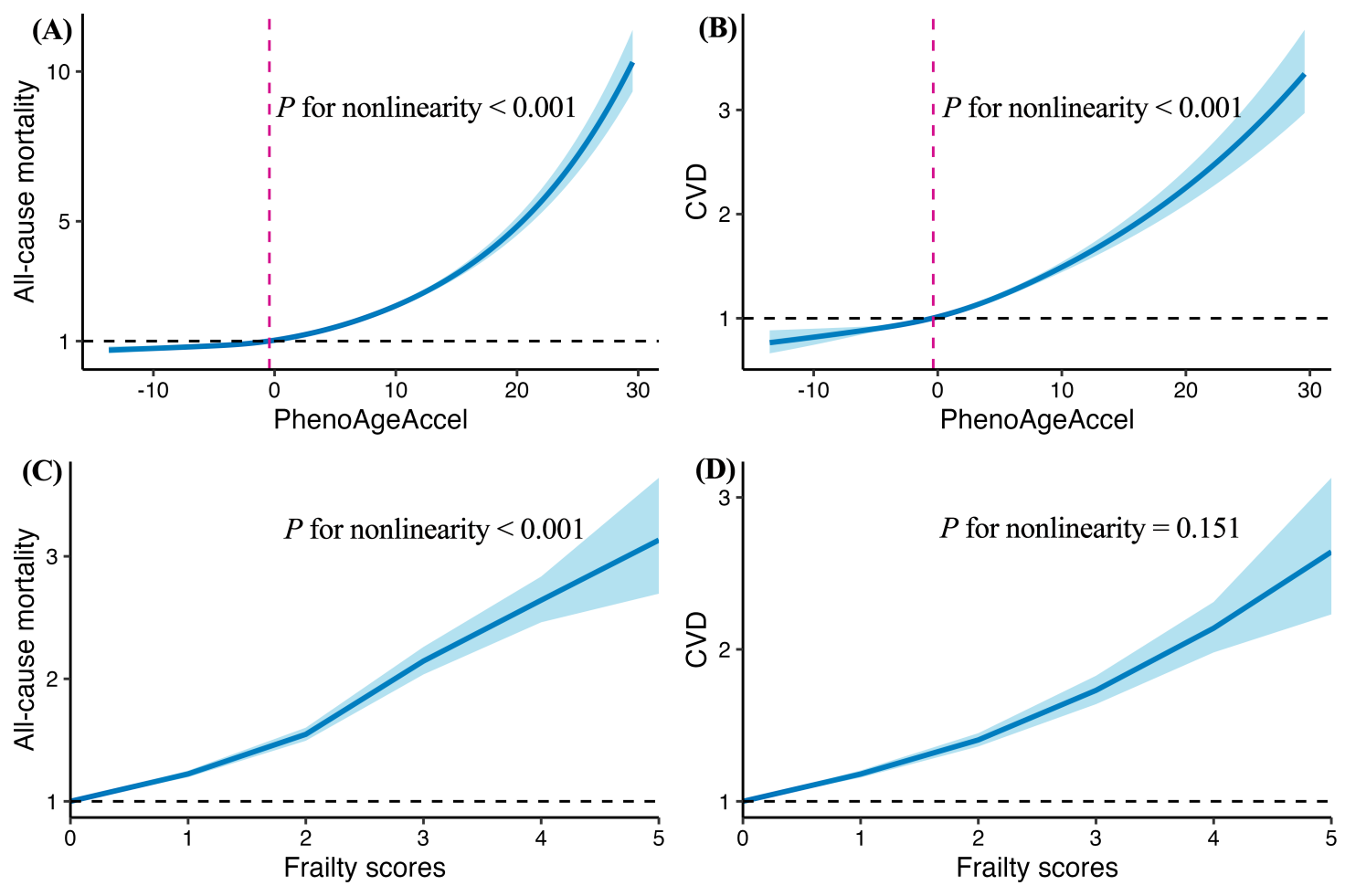
**

**Fig. S4** Restricted cubic splines (RCS) for associations of accelerated biological aging with all-cause mortality and CVD incidence

The models were adjusted for age, gender, BMI, smoking status (never/former/current), sleep status (normal/excess/lack), deprivation index, drinking frequency (< 3 or ≥ 3 times a week), hypertension (yes/no), diabetes (yes/no), hyperlipidemia (yes/no), and renal impairment (yes/no).
